# Uniform Widespread Nuclear Phosphorylation of Histone H2AX Is an Indicator of Lethal DNA Replication Stress

**DOI:** 10.3390/cancers11030355

**Published:** 2019-03-13

**Authors:** Eric Moeglin, Dominique Desplancq, Sascha Conic, Mustapha Oulad-Abdelghani, Audrey Stoessel, Manuela Chiper, Marc Vigneron, Pascal Didier, Laszlo Tora, Etienne Weiss

**Affiliations:** 1 Biotechnologie et Signalisation Cellulaire, UMR 7242, CNRS/Université de Strasbourg, Boulevard S. Brant, 67412 Illlkirch, France; eric.moeglin@unistra.fr (E.M.); desplanc@unistra.fr (D.D.); audrey.stoessel@unistra.fr (A.S.); chiper@unistra.fr (M.C.); marc.vigneron@unistra.fr (M.V.); 2 Institut de Génétique et de Biologie Moléculaire et Cellulaire, 67404 Illkirch, France; conic@igbmc.fr (S.C.); oulad@igbmc.fr (M.O.-A.); laszlo@igbmc.fr (L.T.); 3 Centre National de la Recherche Scientifique, UMR 7104, 67404 Illkirch, France; 4 Institut National de la Santé et de la Recherche Médicale, U1258, 67404 Illkirch, France; 5 Université de Strasbourg, 67404 Illkirch, France; 6 Laboratoire de Bioimagerie et Pathologies, UMR 7213, CNRS/Université de Strasbourg, Route du Rhin, 67401 Illkirch, France; pascal.didier@unistra.fr

**Keywords:** histone variant, H2AX phosphorylation, γ-H2AX, pan-nuclear pattern, monoclonal antibody, cancer cells, cell death, replication stress, chemotherapy, H2AFX gene, knock-out

## Abstract

Phosphorylated histone H2AX (γ-H2AX), a central player in the DNA damage response (DDR), serves as a biomarker of DNA double-strand break repair. Although DNA damage is generally visualized by the formation of γ-H2AX foci in injured nuclei, it is unclear whether the widespread uniform nuclear γ-H2AX (called pan-nuclear) pattern occurring upon intense replication stress (RS) is linked to DDR. Using a novel monoclonal antibody that binds exclusively to the phosphorylated C-terminus of H2AX, we demonstrate that H2AX phosphorylation is systematically pan-nuclear in cancer cells stressed with RS-inducing drugs just before they die. The pan-nuclear γ-H2AX pattern is abolished by inhibition of the DNA-PK kinase. Cell death induction of cancer cells treated with increasing combinations of replication and kinase (ATR and Chk1) inhibitory drugs was proportional to the appearance of pan-nuclear γ-H2AX pattern. Delivery of labeled anti-γ-H2AX Fabs in stressed cells demonstrated at a single cell level that pan-nuclear γ-H2AX formation precedes irreversible cell death. Moreover, we show that H2AX is not required for RS-induced cell death in HeLa cells. Thus, the nuclear-wide formation of γ-H2AX is an incident of RS-induced cell death and, thus, the pan nuclear H2AX pattern should be regarded as an indicator of lethal RS-inducing drug efficacy.

## 1. Introduction

The nucleosome contains two copies of each core histone proteins (H2A, H2B, H3, and H4) and 146 base pairs of superhelical DNA wrapped around this histone octamer. Histone H2AX is a variant of the core histone H2A family and is present in 2–25% of the mammalian nucleosomes deposited on the chromatinized genome, depending on the organism and cell type [1]. Core histone proteins contain N-terminal and C-terminal tails that are sites for post-translational modifications. In contrast to the canonical H2A, H2AX contains a unique Ser-Gln (SQ) motif in its C-terminal tail. The serine residue (S139) located four residues from the C-terminus of H2AX is rapidly phosphorylated upon DNA breakage. This phosphorylated form of H2AX is called gamma-H2AX (γ-H2AX). Phosphorylation of H2AX is one of the earliest events upon DNA double-strand break (DSB) induction, a severe form of DNA damage that leads to apoptosis if unrepaired [2].

The enzymes responsible for the phosphorylation of Ser139 of the H2AX are members of the PI3 kinase family, including ataxia telangiectasia mutated (ATM), AT and Rad-3 related (ATR), and DNA-dependent protein kinase (DNA-PK) proteins [3,4]. Upon DSB induction, one of these kinases phosphorylates H2AX molecules present in the chromatin regions that surround the lesion. The dynamic spreading of H2AX phosphorylation over mega base pairs of chromosomal DNA is a complex process and depends on the interaction of several other actors including MDC1, which binds directly to γ-H2AX for sensing the DSB [5]. In general, accumulation of γ-H2AX at the DSB sites is monitored with specific antibodies, which reveal nuclear foci under the microscope. Moreover, it is well accepted that the number of such γ-H2AX-labeled foci is directly proportional to the number of DSBs and, thus, gives an estimate of the severity of the DNA damage and/or the cytotoxicity of a given genotoxic agent [6].

Although γ-H2AX is generally considered as a biomarker of DSBs, it is widely accepted that γ-H2AX is also a key regulator of the DNA damage response (DDR) when the progression of the DNA replication forks is inhibited [7]. Replication halting, generally referred to as replication stress (RS), generates single-stranded DNA (ss DNA) that is prone to endonuclease cleavage, when the RPA protein levels are exhausted [8]. Fortunately, the serine/threonine kinases ATR and Chk1 counteract global replication fork collapse [9] by delaying the progression of the cell cycle and by promoting DNA repair through the activation of downstream actors via phosphorylation of H2AX. These kinases through their signaling via γ-H2AX constitute the main actors of the replication stress response (RSR) and the inhibition of the function of these kinases with drugs can thus lead to replication catastrophe. Cells can than either recover or die depending on the strength of the RS [10]. Importantly, as cancer cells have already an oncogene induced RS [11] they are more sensitive to additional RS (replication halting + inhibition of RSR) than normal cells, numerous approaches of chemotherapy and radiotherapy have been developed to trigger lethal RS in cancer cells [12]. In these studies, following drug administration and/or heavy ion irradiation γ-H2AX is used as a read-out to measure the generated RS. Whilst γ-H2AX foci formation is considered as a genotoxicity endpoint in most studies, a few recent reports described that a γ-H2AX pattern that suggested widespread uniform phosphorylation of H2AX in the nucleus upon genotoxic treatment. Such widespread and uniform nuclear γ-H2AX phosphorylation (hereafter called pan-nuclear signal) seems to be proportional to the intensity of the induced RS [13,14,15]. In addition, this pan-nuclear γ-H2AX pattern is observed under several RS induction conditions [16,17,18].

Previously, we have shown that in vivo inhibition of the functions of either PCNA or DNA polymerase alpha (which are key components of the replisome) by antibodies, strongly induced RS and the consequent phosphorylation of H2AX in cancer cells [19]. Interestingly, in our study, γ-H2AX showed a clear pan-nuclear pattern in a prominent fraction of the treated cells and these cells died upon prolonged incubation. Thus, our previous study opened the possibility that the specific inhibition of DNA replication can lead to widespread nuclear accumulation of γ-H2AX, which in turn could induce cell death. To better understand the relationship between the widespread pan-nuclear γ-H2AX pattern and cell death following replication stress, first we have generated a specific monoclonal antibody (mAb) against γ-H2AX that recognizes specifically the phosphorylated form of H2AX, but does not cross-react at all with the nonphosphorylated C-terminal tail of H2AX. Next, we have used this new mAb to investigate the fate of cancer cells following treatments with a series of RS-inducing drug combinations. Here, we show that widespread uniform pan-nuclear γ-H2AX phosphorylation pattern represents an ultimate degree of H2AX phosphorylation in the genome that precedes irreversible cell death. In conclusion, our study demonstrates that pan-nuclear γ-H2AX phosphorylation pattern can be used as indicator of drug efficacy for RS-dependent cell death in clinical applications.

## 2. Results

### 2.1. Analysis of the Dynamics of Phosphorylated H2AX with Newly-Generated Monoclonal Antibodies

**Figure 1:**
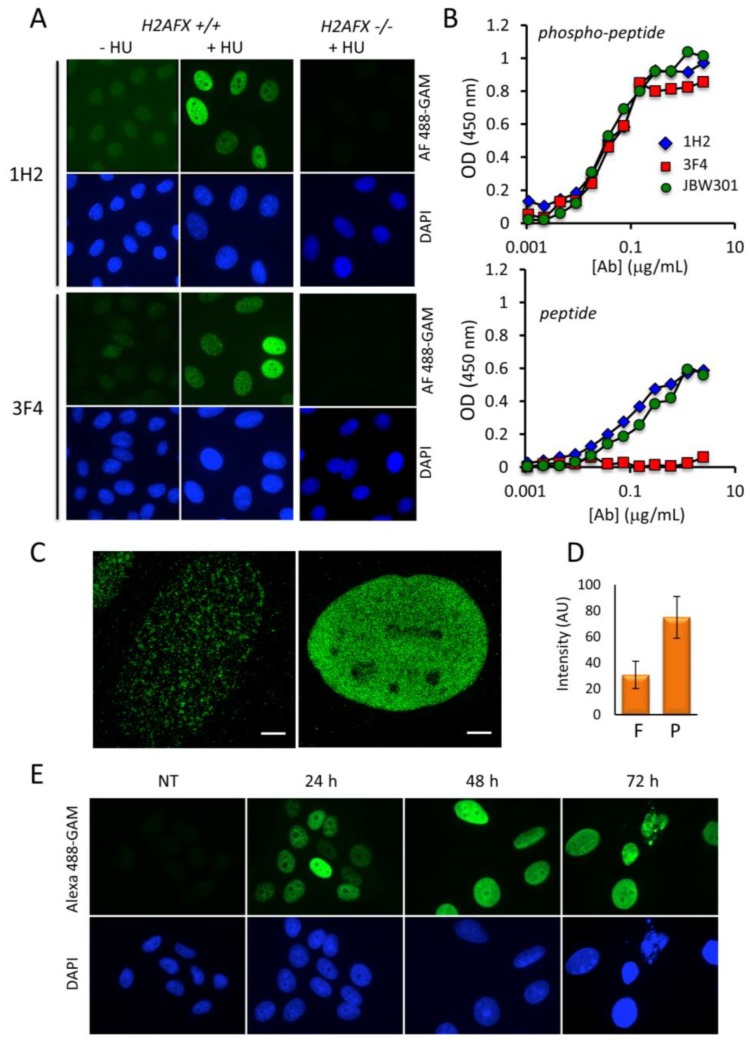
Detection of HU-induced γ-H2AX in HeLa cells with the newly-generated antibodies. (**A**) After treatment with HU for 24 h, either wild type or H2AFX^−/−^ HeLa cells were subjected to analysis by immunofluorescence with a conventional microscope (Materials and Methods). The binding of mAb 1H2 or mAb 3F4 was revealed with Alexa Fluor 488-labeled goat anti-mouse immunoglobulins (green). The nuclei were counterstained with DAPI (blue). Magnification: 630×. (**B**) Analysis of the binding specificity of mAbs 1H2, 3F4 and JBW301 by ELISA. After incubation in the presence of either the phosphorylated (phospho-peptide) or the nonphosphorylated (peptide) peptides corresponding to the C-terminus of H2AX coated on plate, bound mAbs were revealed with HRP-labeled secondary anti-mouse globulins. The curves summarize the data obtained in 2 independent experiments. The color code is indicated. (**C**) The HeLa cells stained with mAb 3F4 shown in A were analyzed by 3D-structural illumination super resolution microscopy (3D-SIM). Typical cells with either foci (left) or nuclear-wide γ-H2AX staining (right) are shown. Scale bar: 4 μm. (**D**) The average nuclear fluorescence intensity of a minimum of 200 HeLa cells harboring either γ-H2AX foci (F) or pan-nuclear γ-H2AX (P) after staining with mAb 3F4 as in A is represented. (**E**) HeLa cells were incubated with HU and fixed 24 h, 48 h, and 72 h post-treatment. γ-H2AX levels were analyzed as in (**A**).

Previously, we have described that the delivery of an anti-PCNA blocking antibody induces huge RS that can be monitored with commercially available anti-γ-H2AX antibodies [19]. In this study, we have observed in many cells the appearance of pan-nuclear γ-H2AX phosphorylation pattern in several RS-induced cells. Since it was unclear whether the observed pan-γ-H2AX signal under the used RS-inducing conditions corresponded only to the phosphorylated forms of H2AX, we decided to generate monoclonal antibodies that would only detect the phosphorylated tail of H2AX and would not cross-react with the nonphosphorylated C-terminal end of H2AX. The counterselection of the obtained hybridoma clones with the nonphosphorylated peptide in parallel with the immunizing γ-H2AX phospho-peptide allowed us to isolate two candidate mAbs (1H2 and 3F4). They react specifically in immunofluorescence (IF) assays with γ-H2AX after treatment of the cells with hydroxyurea (HU), a ribonucleotide reductase (RNR) inhibitor that mainly affects the nucleotide pool availability and thus induces RS [20] (Figure 1A). Following IF detection of γ-H2AX, two IF staining patterns were observed after HU treatment: (i) most cells displayed a punctuated staining generally called RS foci, and (ii) a fraction of the cells showed an intense widespread nuclear pan-γ-H2AX staining (Figure 1A,C), as previously observed [19]. To verify whether the newly-generated mAbs bind only to γ-H2AX, we created a HeLa cell line in which the *H2AFX* gene, coding for H2AX protein, has been invalidated using the CRISPR/Cas9 technology (Materials and Methods). As expected, no γ-H2AX signal was detected in this *H2AFX*^−/−^ cell line after HU treatment (Figure 1A), indicating that both mAbs specifically recognize H2AX. Similar results were obtained with the commercial anti-γ-H2AX mAb JBW301 (Supplementary Figure S1A). Next, we analyzed whether the raised mAbs were specific to the phosphorylated form of H2AX. In depth analysis by ELISA showed that, while mAb1H2, mAb3F4 and mAbJBW301 all recognized the phosphorylated H2AX peptide, only mAb3F4 did not cross-react with the nonphosphorylated epitope peptide even at elevated mAb concentrations (i.e., >0.1 μg/mL) (Figure 1B).

In addition, when full length 1H2 and 3F4 mAbs were introduced by electroporation in the cytoplasm of untreated HeLa cells [21], we observed that 1H2 was translocated into to the nucleus in a piggybacked fashion over time, but mAb3F4 remained in the cytoplasm under these conditions (Supplementary Figure S1B). This demonstrates that mAb1H2 can bind the nonphosphorylated C-terminus of de novo synthesized H2AX with which it gets piggybacked to the nucleus (see also Conic et al. [22]). In contrast, mAb 3F4 was not able to bind to its target in the cytoplasm, as H2AX gets only phosphorylated in the nucleus. Consequently, mAb3F4 was not piggybacked in the nucleus, further demonstrating the exquisite specificity of mAb 3F4 against γ-H2AX. Thus, as the 3F4 antibody turned out to be the most specific antibody recognizing only the phosphorylated form of H2AX in vitro and in cells, it was used in all further experiments.

To characterize the precise intranuclear distribution of γ-H2AX we used mAb 3F4 by 3D structural illumination super resolution microscopy (3D-SIM). As described by Natale et al. [23], the foci observed at high resolution correspond to clusters of nanofoci that are present in the majority of the HeLa cells treated with HU for 24 h (Figure 1C). In the pan-nuclear stained nuclei, the nanofoci were almost contiguous suggesting that a very large fraction of the histone H2AX is phosphorylated in the chromatin of these cells. Next, we quantified the intensity of fluorescence of the pan-nuclear stained nuclei versus those displaying only foci (see Figure 1A) and found that γ-H2AX levels in the pan-nuclear cells are increased by a factor of 2 to 3 (Figure 1D). To determine whether this enhancement of H2AX phosphorylation is correlated with the appearance of pan-nuclear stained cells, we performed time course IF experiments and observed that almost all nuclei were intensively stained after a 48 h treatment with HU (Figure 1E and Supplementary Figure S2A). This suggests that pan-nuclear distribution of γ-H2AX corresponds seemingly to saturated levels of H2AX phosphorylation, which are reached when the HeLa cells are continuously incubated with HU. Our FACS analyzes indicated that these cells correspond to S phase-arrested cells (Supplementary Figure S2B), as previously described [20]. Interestingly, the size of the nuclei of the pan-nuclear stained cells was increased when compared to that of untreated cells Figure 1E). Finally, the number of cells that could be observed at 72 h of incubation with HU was below to that of the seeded cells, suggesting that saturated widespread nuclear γ-H2AX phosphorylation may represent a feature of those cells that will ultimately die. In addition, the increase of γ-H2AX was also detectable by Western blot analysis (Supplementary Figure S2C), indicating that the phosphorylation of H2AX protein accumulates over time upon Chk-1 protein activation by phosphorylation (Supplementary Figure S2C). In conclusion, we have developed a monoclonal antibody (mAb 3F4) that allows the analysis with confidence the dynamic phosphorylation of H2AX that accumulates as widespread γ-H2AX upon sustained RS.

### 2.2. The Appearance of the Pan-Nuclear γ-H2AX Pattern Is Time and Drug-Dependent

**Figure 2:**
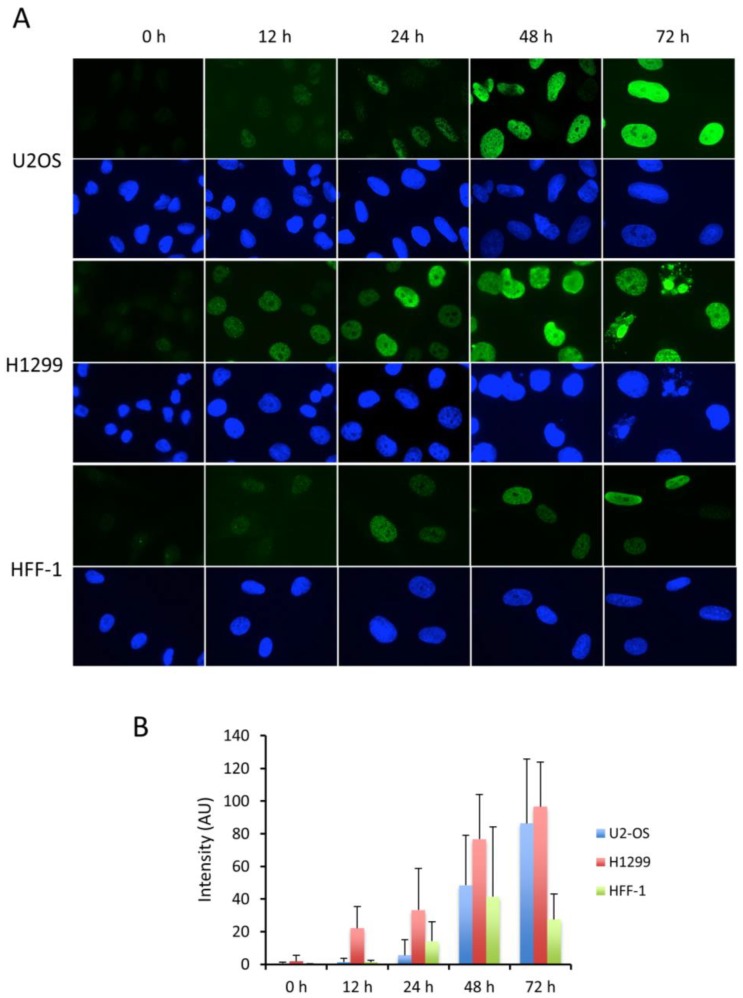
Dynamics of γ-H2AX pattern formation in U2OS, H1299 and HFF-1. The cells shown in (**A**) were treated with HU as in Figure 1. The accumulation of γ-H2AX overtime was revealed with mAb 3F4 either by immunofluorescence microscopy (**A**) or by measuring the fluorescence intensity of a minimum of 150 nuclei per condition recorded from three different experiments (**B**). The pictures shown in (**A**) correspond to typical fields of the observed cells. Magnification: 630×. In (**B**) are represented the average values of the measured nuclear fluorescence intensities after subtraction of the background mean values recorded at 0 h.

**Figure 3:**
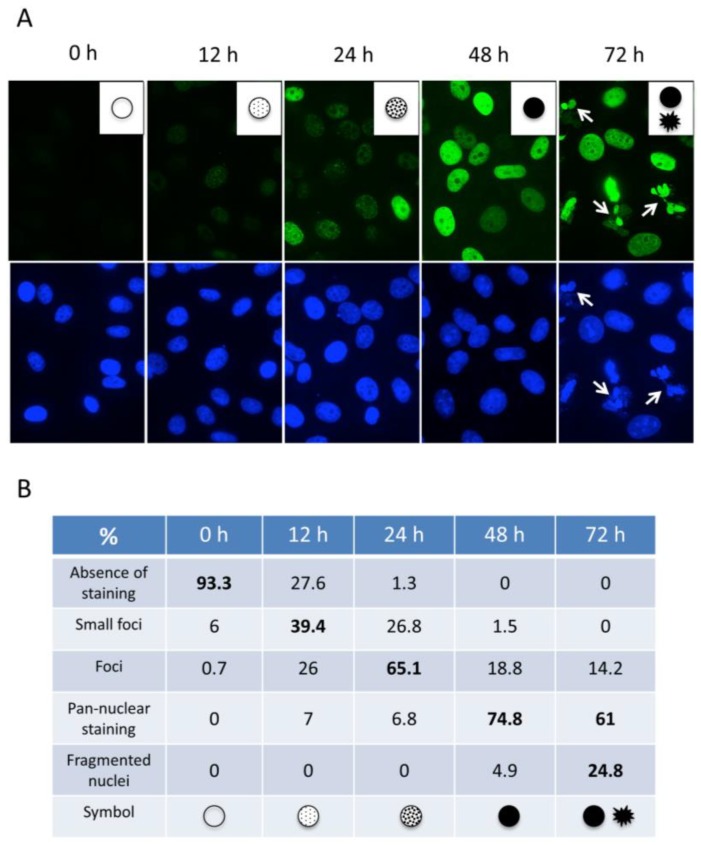
Dynamics of γ-H2AX formation in HeLa cells following treatment with HU. (**A**) Typical patterns of γ-H2AX staining observed during the analysis with mAb 3F4 by immunofluorescence are shown and represented as symbols (insets). Cells with fragmented nuclei at 72 h post-treatment are indicated (white arrows). Magnification: 630×. The assignment of the symbols that correspond to the major characteristic γ-H2AX patterns observed in (**A**) is shown (**B**). Up to 160 nuclei recorded from three independent experiments at each time point were analyzed to calculate the percentages.

To assess whether the pan-nuclear γ-H2AX pattern can also be observed in other cancer cells upon continuous HU-induced replication stress, we tested the behavior of U2OS (p53-positive) and H1299 (p53-negative) cells, in parallel with nontransformed HFF-1 fibroblasts. As seen in HeLa cells, in both cancer cell types, which were almost completely lacking γ-H2AX staining before the treatment, we observed at the earliest time point (12 h) of HU treatment the appearance of γ-H2AX foci and progressively over time the nuclei of treated cells became pan-γ-H2AX stained (Figure 2A and Supplementary Figure S3A). Interestingly, the pan-nuclear phenotype was clearly detectable at 24 h post-treatment in H1299 cells, whereas only foci were visible in the U2OS cells at this time point. This might be linked to the faster division rate of the H1299 cells. The staining of γ-H2AX in the nontransformed HFF-1 fibroblast cells performed in parallel was much less pronounced and only a few cells became pan γ-H2AX positive at the end of the incubation period, indicating that “normal” cells are much more resistant to HU treatment. The sequential accumulation of γ-H2AX in the nuclei of the different cell types over time was also quantified by fluorescence intensity measurements (Figure 2B) and confirmed by Western blotting (Supplementary Figure S3A). In addition, most of the pan-γ-H2AX stained U2OS and H1299 nuclei were slightly enlarged upon incubation with HU during 48 h. This nuclear enlargement was barely observed with the HFF-1 cells, suggesting that in the tested cancer cells the large number of RS-induced DNA breaks provoke a general chromatin decondensation. Interestingly, a significant part of the H1299 nuclei formed smaller multinucleated pan-γ-H2AX stained structures at the 72 h time point suggesting that these cells may undergo apoptosis following genome-wide chromatin decondensation. To quantify and better characterize the observed phenotypic changes during HU treatment in the cells, we standardized the microscopic assay and converted at each time point the observed major characteristic γ-H2AX patterns into symbols (Figure 3A) to perform statistical analyzes of the recorded nuclei (Figure 3B). This analysis demonstrated that in HeLa cells a 12–24 h HU treatment induces mainly γ-H2AX foci, while 48–72 h HU treatments result mainly in pan-nuclear γ-H2AX patterns, giving rise to fragmented nuclei at the longest treatment (Figure 3B).

**Figure 4:**
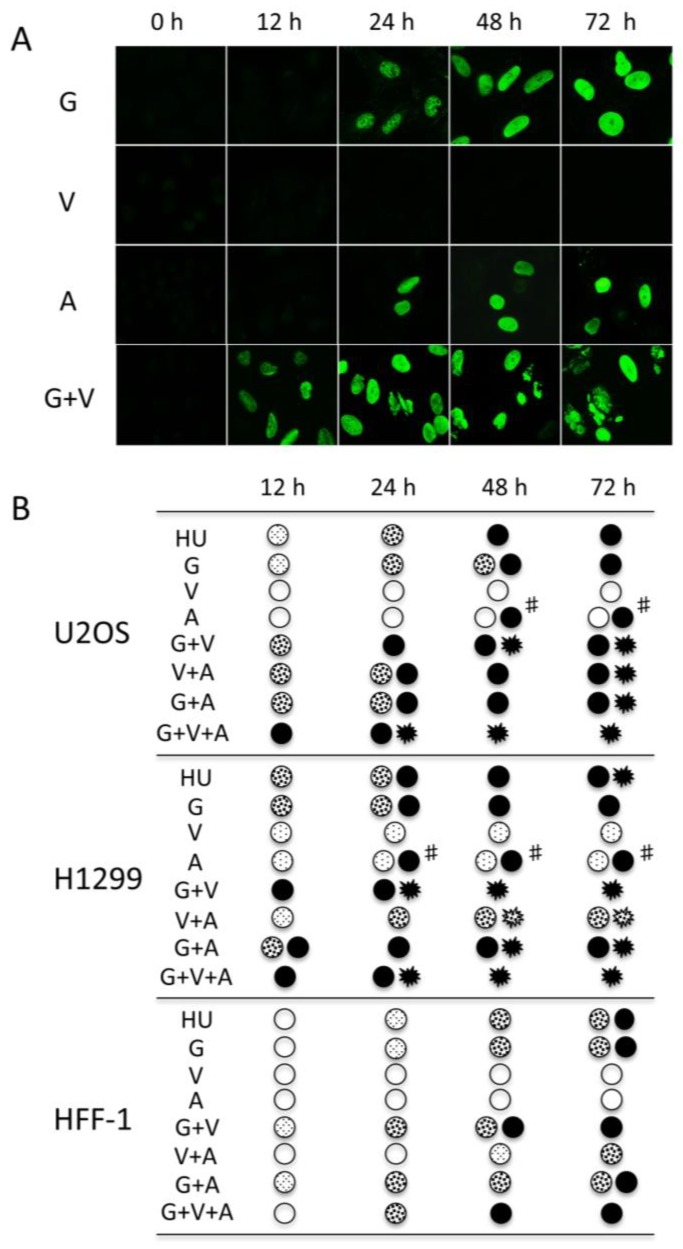
Dynamics of γ-H2AX formation in U2OS, H1299, and HFF-1 cells following treatment with drug combinations. (**A**) Typical patterns of γ-H2AX staining observed during the time point analysis of U2OS cells by immunofluorescence with mAb 3F4 after treatment with either G, V, A, or G+V are shown. The corresponding cells after DAPI staining are shown in Supplementary Figure S3. Magnification: 630×. (**B**) U2OS, H1299, and HFF-1 cells were treated with the indicated drugs or drug combinations. At 12 h, 24 h, 48 h, and 72 h post-treatment, the nuclei were stained with mAb 3F4 and the γ-H2AX patterns were scored as indicated in the legend of Figure 3. A minimum of 120 cells from three independent experiments were analyzed in each condition. #, only a fraction of the cells within the analyzed population showed the indicated pattern.

Next, we asked using this standardized assay whether the saturated widespread pan-γ-H2AX staining obtained after prolonged HU treatment can also be observed with other drugs that induce RS. We analyzed the fate of γ-H2AX upon treatment of U2OS cells with gemcitabine (G), VE-821 (V), and AZD-7762 (A) over a 72 h time period. G targets specifically the RNR (similar to HU), V is a potent inhibitor of the ATR protein and A blocks specifically the Chk1 kinase [24]. Treatment with G alone led to the same result as obtained with HU, i.e., to the appearance of essentially pan-nuclear-stained cells after 48 h of incubation. The use of V alone had almost no effect on H2AX phosphorylation and only of a fraction of the cells incubated with the Chk1 inhibitor (A) was γ-H2AX-positive after the prolonged incubation (Figure 4 and Supplementary Figure S3B). However, when G and V were combined for the treatment, strong staining of γ-H2AX was observed already after 12 h of incubation and the pan-nuclear stained nuclei were easily detectable at the 24 h incubation time (Figure 4A). Remarkably, a fraction of the positive nuclei were fragmented upon 48 h of treatment, as seen with H1229 cells after 72 h HU treatment (Figure 2A). Since it is well known that inhibition of ATR blocks RS-induced DNA damage repair and enhances thereof the RS effect of G [25], our results obtained with G+V suggest that the pan-nuclear staining of γ-H2AX is an indication of excessive RS that cannot be rescued before mitosis and which thus leads to mitotic catastrophe and cell death. 

Next, we tested the combinations of V and A (V+A [13]), G and A (G+A [26]) and also the three drugs together (G+V+A). As shown in Figure 4B, the treatment with these different combinations triggered nuclear-wide phosphorylation of H2AX after overnight incubation with the cells. The G+V+A was the most potent mixture to reach rapid pan-nuclear accumulation of γ-H2AX. In addition, in this case, only fragmented nuclei with intense γ-H2AX staining remained attached to the culture dish after 48 h of treatment. This suggests the pan-nuclear γ-H2AX phenotype is not a simple signature of RS, but rather a dynamic result of the intensity of the RS that is timely regulated with regard to the number of different RS-inducing drugs. A similar analysis was also performed with H1299 and HFF-1 cells (Figure 4B). We found that the H1299 cells reacted slightly faster than the U2OS cells to the different drug combinations. For instance, H1299 cells were almost all pan-nuclear positive following the treatment with G+V or G+A following 12 h. However, their treatment with the V+A mixture did not trigger pan-nuclear accumulation of γ-H2AX during the 72 h treatment period. This could be due to the absence of p53 protein expression in this cell line since a similar result was obtained with the HeLa cells that are also p53-deficient (Supplementary Figure S4A). There was a considerable difference in γ-H2AX accumulation between the cancer cells and the nontransformed HFF-1 cells, independently of the drugs or drug combinations used. These results are in good agreement with the observations suggesting that normal cells are less sensitive to RS-inducing drugs than cancer cells [13], likely due to their intrinsic lower levels of endogenous RS. 

We also analyzed in U2OS and H1299 cells the effect on γ-H2AX formation of two classical genotoxic drugs, campthotecin (CPT), and epirubicin (EPI), that are daily used in the clinic and which target topoisomerase I and II, respectively. The presence of these drugs in the culture medium led to formation of numerous γ-H2AX foci after 24 h of incubation and upon prolonged treatment, a pan-nuclear staining of γ-H2AX was detected in a large percentage of the treated cells (Supplementary Figure S5). This was particularly evident in H1299 cells similarly to the experiments described above. In conclusion, our results together confirm that the nuclear wide phosphorylation of H2AX is a general mark of intense RS mediated by drugs that inhibit DNA and RNA synthesis. 

To test whether human multidrug-resistant promyelocytic HL60R leukemia cells [27] would react to the above tested RS-inducing drugs with a similar pan-γ-H2AX pattern, we used the above-described G, V, and A drugs and their combinations. Although HL60R cells are resistant to daunorubicin or etoposide treatments, two well described drugs that suppress topoisomerase II activity in mammalian cells, they were as sensitive as the H1299 cells to the different mixtures of G, V, and A drugs (Supplementary Figure S4A). However, the fluorescence in the pan-nuclear stained HL60R cells was not as bright as that obtained with the other tested cancer cells, likely due to the fact that their nucleus diameter was increased by a factor of 2–3 after 48 h treatment with G or with all combinations containing G (Supplementary Figure S4B). This particular feature has also been observed in a previous study after intracellular delivery of an anti-PCNA blocking antibody [19]. Overall, our data indicate that the pan-nuclear γ-H2AX phenotype can be observed in a variety of cancer cells following prolonged intense RS induction. 

### 2.3. The Accumulation of the Widespread Pan-Nuclear γ-H2AX Pattern Is a Result of DNA-PK Hyperactivation

**Figure 5:**
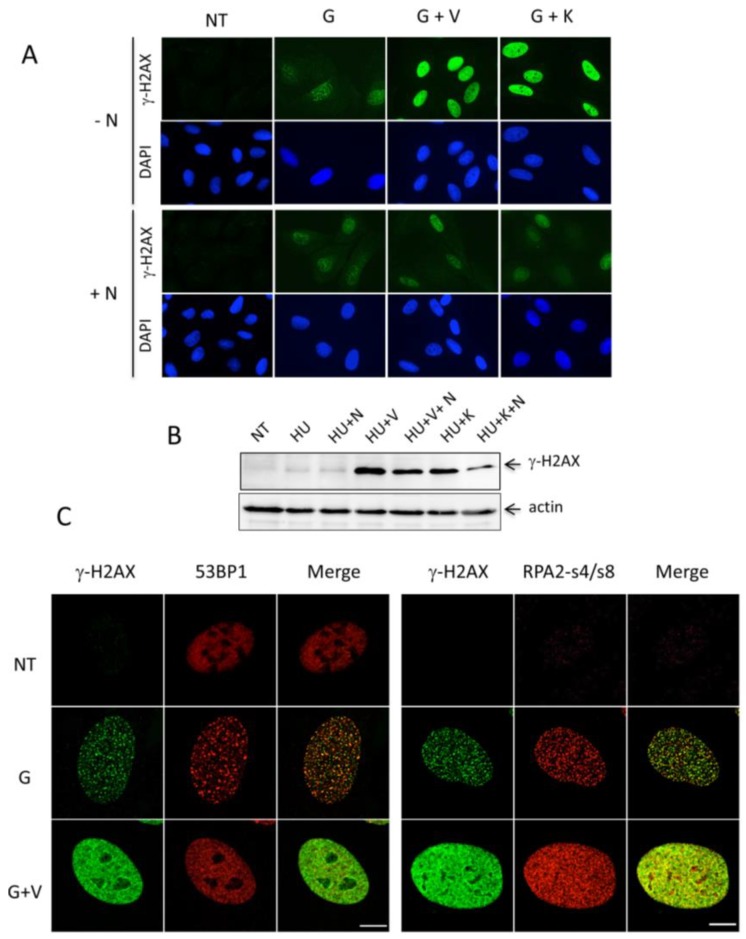
Involvement of DNA-PK in γ-H2AX formation and analysis of 53BP1 and phospho-RPA S4/S8 levels upon induced replication stress. (**A**) U2OS were treated with G, G+V, or G+K in the presence (+) or the absence (−) of the DNA-PK inhibitor N. 24 h post-treatment, the cells were fixed and analyzed for γ-H2AX formation as described in the legend of Figure 2. The micrographs show typical fields of cells observed by immunofluorescence from three independent experiments. Magnification: 630×. NT, nontreated. (**B**) Analysis of the γ-H2AX levels by Western blotting. After treatment of U2OS cells with the indicated drugs during 24 h, crude cell extracts (30 μg) were subjected to Western blot analysis. The γ-H2AX polypeptides were revealed with mAb 3F4. β-actin was used as a loading control. NT, nontreated. (**C**) Analysis of 53BP1 and phospho-RPA S4/S8 levels by confocal immunofluorescence. U2OS cells treated with G or G+V for 24 h were incubated after fixation with anti-53BP1 or anti phospho-RPA (RPA2-s4/s8) polyclonal antibodies (Materials and Methods). The cells were costained with mAb 3F4. Bound antibodies were revealed with fluorescent secondary anti-mouse (green) or anti-rabbit (red) globulins. The pictures show representative nuclei recorded from 120 individual cells observed under the confocal microscope. Scale bar: 10 μm. NT, nontreated.

It is well established that the C-terminus of H2AX can be phosphorylated by either ATM, ATR or DNA-PK kinases in response to DNA replication fork stalling and/or DNA breakage [9]. The above results show that the inhibition of ATR with the chemical inhibitor V leads to strong nuclear γ-H2AX formation. To examine if either ATM or DNA-PK are activated upon incubation of the cells with G and V, we cotreated U2OS cells for 24 h with the selective chemical inhibitors KU-60019 (K; ATMi) or NU-7441 (N; DNA-PKi), in addition to G or G+V. The distribution and intensity of induced γ-H2AX was monitored under the microscope. Whilst either G or K alone did not trigger any pan-nuclear modification of H2AX as expected, the addition of K to G led to a strong γ-H2AX signal, almost equivalent to that observed with the G+V mixture (Figure 5A). Interestingly however, when G-, G+V-, or G+K-treated cells were coincubated with N, no pan-nuclear γ-H2AX staining was observed and only foci were identified. As N is a DNA-PK inhibitor, these results strongly suggest that DNA-PK is responsible for the spreading of the H2AX phosphorylation over the whole nucleus in the absence of N (Figure 5A, compare –N and +N panels). This result was confirmed by Western blotting (Figure 5B) and by measuring the nuclear fluorescence intensities (Supplementary Figure S6A) after cotreatment of U2OS cells with HU and the different kinase inhibitors mentioned above. Thus, pan-nuclear γ-H2AX observed after intense RS results clearly from DNA-PK activation, as proposed also by Meyer et al. [17]. 

Since DNA-PK enables mainly nonhomologous end-joining (NHEJ) repair following DNA double strand breakage (DSBs), we investigated whether the 53BP1 protein, which is actively involved in the DSB repair process [28], would colocalize with the widespread nuclear γ-H2AX pattern under G or G+V treatments. Figure 5C shows typical cells stained with an anti-53BP1 antibody after analysis by confocal microscopy following treatment for 24 h with either G or G+V. The 53BP1 protein, which is present in the whole nucleus of the untreated cells, is detected as bright foci structures after G treatment. Upon G+V treatment, a wealth of smaller 53BP1 foci that are less bright and almost contiguous were observed, in parallel with pan-nuclear γ-H2AX formation. However, the analysis of the overlays using the Pearson’s correlation coefficient [29] shows that ~60% of the γ-H2AX and 53BP1 molecules colocalize under these conditions (Figure 5C, left panel). We also analyzed the distribution of phosphorylated RPA32 Ser4/Ser8, another recently described target of DNA-PK in response to fork stalling [30]. Whereas almost no phosphorylated RPA32 was detected in the nontreated cells, bright individual and nuclear-wide foci were apparent upon G or G+V treatments at 24 h, respectively (Figure 5C, right panel). As observed with 53BP1, the signals of phospho-RPA32 and γ-H2AX did clearly overlap (up to 70% of calculated colocalization), suggesting that pan-nuclear γ-H2AX colocalize with phosphorylated RPA32 Ser4/Ser8 at the stalled forks. 

To determine whether these phosphorylation events were accompanied with extensive DNA breakage, we extracted the genomic DNA of G- or G+V-treated cells and performed agarose gel analysis. The DNA extracted from the cells at 24 h post-treatment (incubation time of the cells analyzed by confocal microscopy) migrated at a single band (Supplementary Figure S6B). However, a faint smear was detectable with the G+V sample after 48 h of incubation and numerous cuts were visible at 72 h post-treatment. These results were confirmed by single cell gel electrophoresis experiments and Comet assays (Supplementary Figure S6C). Together, these data suggest that the formation of pan-nuclear γ-H2AX pattern is not a marker of DSBs, but very likely a signature of intense RS with stalled forks that activate the DNA-PK kinase.

### 2.4. Pan-Nuclear γ-H2AX Staining Is Correlated with the Loss of Cell Viability

**Figure 6:**
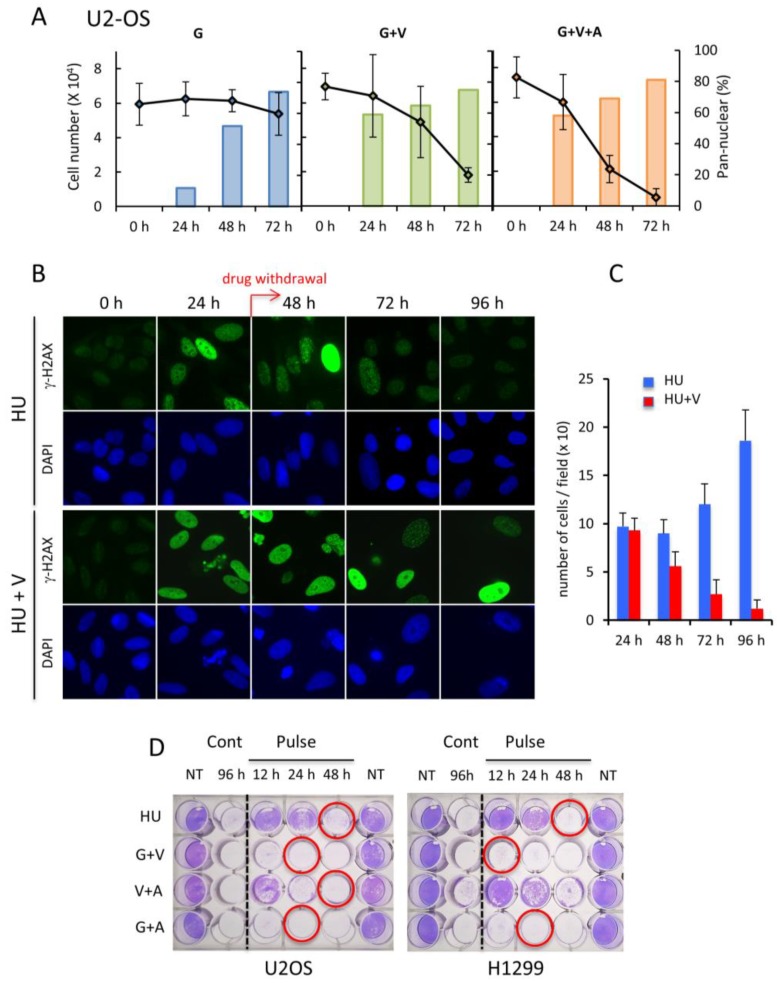
Cell death induction upon formation of widespread pan-nuclear γ-H2AX. (**A**) Time course analysis of the percentage of pan-nuclear γ-H2AX-stained U2OS cells after treatment with G, G+V, or G+V+A. The graphs correspond to the number of cells that were bound to the support in parallel experiments. A minimum of 150 cells were analyzed in each case. (**B**) U2OS cells were incubated with either HU or HU+V. Twenty-four hours post-treatment, the drugs were removed by replacing the culture medium with fresh medium (pulse treatment). The pictures show the γ-H2AX levels of representative nuclei monitored by immunofluorescence before (0 h) or after the treatment (24 h) or after 1 (48 h), 2 (72 h), or 3 (96 h) days of drug withdrawal. Magnification: 630×. The calculated number of cells remaining attached to the coverslips under these conditions is represented in (**C**). (**D**) Viability assays of pulse-treated U2OS or H1299 cells. Equal numbers of seeded cells were either continuously (Cont) or pulse- (Pulse) treated with the indicated drug mixtures. 96 h post-incubation, the cells were fixed and stained with crystal violet (Materials and Methods). The red circles correspond to the time of treatment which triggers in each condition mainly widespread pan-nuclear γ-H2AX staining as shown in Figure 4. NT, nontreated.

The drugs used in this study are toxic for the cells and are used in the clinic for promoting cancer cell death. Numerous studies have shown that the formation of hundreds of γ-H2AX foci could be an indication of RS-induced cell lethality [6], but it remains unclear if pan-nuclear γ-H2AX is directly associated with this loss of cell viability. By counting the U2OS cells remaining alive in the containers after treatment with either G, G+V or G+V+A and by analyzing in parallel the pattern of γ-H2AX, we found that the increased proportion of pan-nuclear stained cells over time (as already shown in Figure 2 and Figure 4) was accompanied with a significant drop of the number of cells remaining attached to the culture dish. This was particularly the case when testing the drug combinations that promote pan-nuclear accumulation of γ-H2AX after 48 h of incubation (Figure 6A). The same effect was observed with the H1299 cells after a similar treatment (Supplementary Figure S7A). Because pan-nuclear γ-H2AX pattern was hardly detectable in HFF-1 cells even after incubation with G+V for 72 h, and as the number of cells remained constant over time, we concluded that cancer cells might acquire a pan-nuclear γ-H2AX accumulation phenotype before cell death. Thus, pan-nuclear γ-H2AX staining could be an indication of cell death initiation. 

To test this hypothesis, we performed pulse drug administration experiments and analyzed if a short incubation of the cells with drugs that promote pan-nuclear γ-H2AX staining would be sufficient to kill them. We incubated U2OS cells with either HU or HU+V for 24 h (that lead to foci or pan-nuclear γ-H2AX, respectively) and recorded the γ-H2AX pattern as well as the cell number after drug withdrawal for three days (Figure 6B). The cells treated with HU alone did not show any γ-H2AX staining after the third day of HU release. In addition, the number of cells in the wells was higher at 72 h after drug withdrawal than that calculated at the time of drug withdrawal (Figure 6C), indicating that the cells can recover from focal γ-H2AX formation. In contrast, upon incubation with HU+V for 24 h and subsequent incubation without drug for 72 h, almost all cells that remained alive the third day after drug withdrawal were pan-nuclear γ-H2AX positive and their number was far lower than the initial number of the treated cells. The fact that H2AX phosphorylation can be resolved after mild RS (foci formation) and not be modified following intense RS (pan-nuclear distribution) was confirmed by Western blot analysis (Supplementary Figure S7B). These results together suggest that the widespread pan-nuclear accumulation of γ-H2AX corresponds to a point of no return for the U2OS cells to a normal state, and that cell viability is compromised when the pan-nuclear γ-H2AX pattern is detectable. Loss of cell viability was observed after pulse treatment of both U2OS and H1299 cells with the G+V, V+A, and G+A combinations that promote pan-nuclear g-H2AX formation (Figure 6D). Together, these results indicate that detection of pan-nuclear γ-H2AX might be a convenient read-out of the efficacy of RS-inducing drugs to trigger cell death.

Because the saturated pan-nuclear γ-H2AX pattern in heavily RS-injured cells seems to be paralleled with initiation of cell death, we tested whether the accumulation of this phosphorylation is required for the death processing. By comparing the survival rate of the *H2AFX^−/−^* HeLa cells to that of the wild type HeLa cells after pulse treatment with different drug combinations, we could not see any differences in cytotoxic effect, nor in survival rate (Supplementary Figure S8A). Similar results were obtained when comparable experiments were done with wild type and *H2AFX^−/−^* HEK293 cells. Moreover, as shown in Supplementary Figure S8B, the number of killed U2OS and H1299 cells did not vary when the treatments were performed in the absence or presence of the DNA-PK inhibitor N (that inhibits the formation of pan-nuclear γ-H2AX; Figure 5A). All together these data indicate that pan-nuclear γ-H2AX formation is not prerequisite for drug-induced cell death, but it may rather correspond to a feature of stressed cells in which DNA repair is overwhelmed and that are subsequently undergoing death [31].

### 2.5. Pan-Nuclear γ-H2AX Pattern Is a Signature of Induced Cell Death and Thus of Lethal Replication Stress

**Figure 7:**
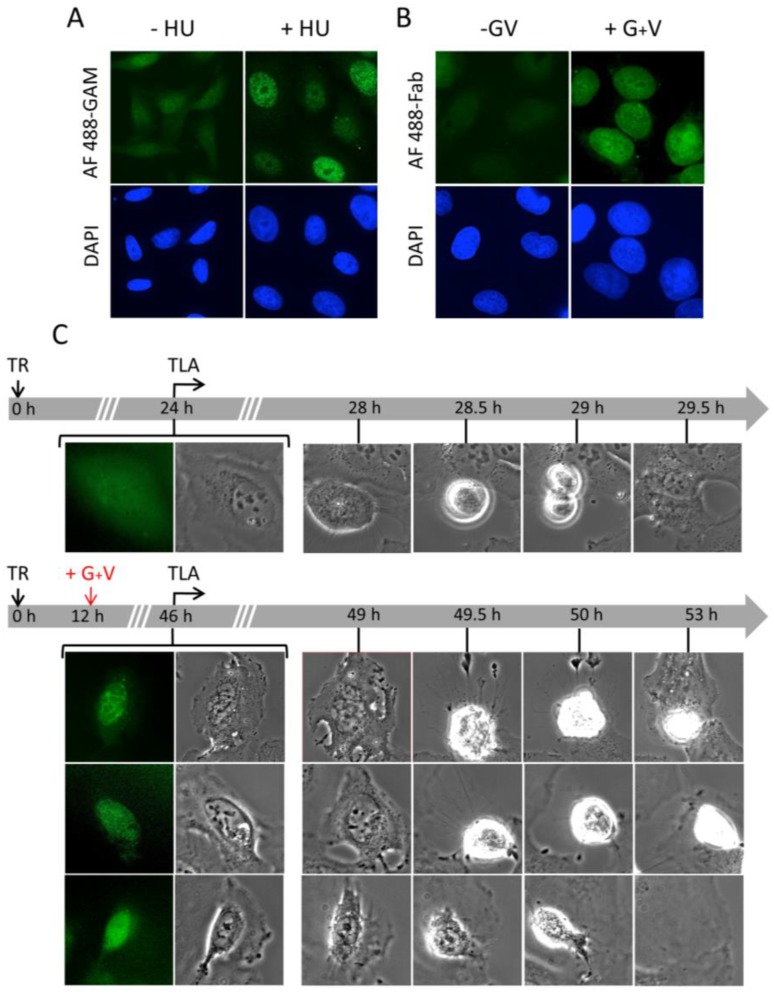
Time-lapse monitoring of γ-H2AX formation in U2OS cells transduced with 3F4 Fabs. Nonlabeled (**A**) or Alexa Fluor 488-labeled (**B**) 3F4 Fabs were delivered by electroporation to U2OS cells. 24 h post-transduction, the cells were treated with either HU (**A**) or G+V (**B**). After 48 h of HU treatment for 48 h or G+V treatment for 24 h, the cells were fixed and analyzed by immunofluorescence microscopy. The pictures show typical fields of the transduced cells observed under the microscope. The non-labeled Fabs were revealed with Alexa Fluor 488-labeled goat anti-mouse immunoglobulin (AF 488-GAM) and the Alexa Fluor 488-labeled Fabs (AF 488-Fab) were visualized without an additional processing. The nuclei were counterstained with DAPI. (**C**) Analysis of the cell fate by time-lapse microscopy following transduction. After transduction (TR), the U2OS cells were either nontreated (upper panel) or treated with G+V (lower panel) as indicated and subjected to time-lapse monitoring. Phase-contrast images of several fields were taken every 30 min during approximately 8 h. The micrographs show representative individual cells of a minimum of 50 cells from three independent experiments recorded by fluorescence and phase-contrast at the beginning of the time-lapse analysis (TLA) and, subsequently, by phase-contrast. Numerous cell division events within the population of the nontreated transduced cells were visible at approximately 30 h post-transduction (upper panel). In the presence of G+V, the cells did not divide and some rounded up and became floating after approximately 50 h of incubation (lower panel). Magnification: 400×.

To demonstrate that the cells with a saturated pan-nuclear γ-H2AX phenotype do not survive, we took advantage of the possibility to deliver labeled antibodies or Fab fragments into living cells [22,32] to follow the fate of those cells with pan-nuclear accumulation of γ-H2AX. Fabs correspond to the antibody arms that encompass the antibody binding capacity. They are obtained by cleavage of the antibody hinge region with papain protease (Materials and Methods). Experiments performed with unlabeled 3F4 Fab fragments, which do not bind to the nonphosphorylated C-terminal H2AX peptide as probed by ELISA (Supplementary Figure S9), showed that they accumulate in the nucleus of U2OS cells upon treatment with HU for 48 h (Figure 7A). We obtained the same results when Alexa Fluor 488-labeled Fabs were used to transduce U2OS cells sensitized with G+V for at least 24 h (Figure 7B). Notably, under these conditions, the fluorescently labeled Fabs were homogeneously distributed in the nuclei as observed above by classical immunofluorescence. We have taken this condition of treatment to follow the fate of individual transduced cells by time-lapse microscopy over a period of 7 h after a treatment with G+V for 34 h. Expectedly, this treatment triggered the formation of pan-nuclear γ-H2AX in most of the cells as visualized with the localization of the labeled Fabs that were present in the nuclei at the beginning of the time-lapse analysis (Figure 7C). Within the population of flat cells that were bound to the culture dish, fragmented nuclei were visible (Figure 7C, lower panel). During the prolonged incubation, about half of the pan-nuclear γ-H2AX-positive cells rounded up and some of them detached from the support during the bright field microscopy analysis (Video S2). This phenomenon corresponds to cell death and is generally observed when cells undergo apoptosis. In contrast, when the G+V treatment was omitted, the fluorescent Fabs were detected both in the nucleus and in the cytoplasm (no re-localization due to the absence of γ-H2AX formation) and they continued to divide (Figure 7C, upper panel and Video S1). Together, these results confirm that the transduced 3F4 Fabs are not cytotoxic by themselves and that they are bound to nuclear-wide γ-H2AX formed in cells that will die. Hence, the widespread nuclear phosphorylation of H2AX is an indication of lethal RS.

## 3. Discussion

This study became possible because we have generated our own mAb, which allowed the specific detection and tracking of the phosphorylation of H2AX. When analyzing the γ-H2AX recognition specificity of the monoclonal antibodies produced by our growing hybridomas, we found that only a few of them were truly γ-H2AX phosphopeptide-specific. Moreover, we have carried out a test that has never been reported before for analyzing the cross-reactivity of full-length antibodies recognizing nuclear phospho-epitopes under physiological conditions (Supplementary Figure S1B). Note that full-length mAbs (150 kDa) enter the nucleus only if they are bound to their neosynthesized targets in the cytoplasm and are piggybacked to the nucleus with their targets [21,22]. The fact that after electroporation full-length mAb 3F4 remained in the cytoplasm, where H2AX is neosynthesized, but is not phosphorylated, clearly indicated that mAb 3F4 did not recognize the nonphosphorylated C-terminus of H2AX. If mAb 3F4 would have recognized the nonphosphorylated H2AX, it would have been piggybacked with de novo synthesized H2AX into the nucleus, but this was not the case (Supplementary Figure S1). This clearly indicates that the variation of γ-H2AX levels detected with 3F4 mAb by IF throughout the study are due to DNA damage. It is conceivable also that commercially available anti-γ-H2AX reagents do cross-react to some extent with nonphosphorylated H2AX [33]. The above described electroporation test could thus be used in the future to which extent these commercial antibodies cross-react with the H2AX. In addition, the observation that electroporated 3F4 Fab (50 kDa), which can freely diffuse to the nucleus, was exclusively relocalized in the nucleus upon drug treatment, indicating that this antibody is a unique tool for monitoring in a high-throughput manner the exquisite kinetics of γ-H2AX in either live or fixed cells exposed to DNA-damaging agents [34,35]. Interestingly, we never observed any effect of the delivered high amount of Fabs on γ-H2AX formation/spreading soon after drug application, nor a delay of formation of γ-H2AX in the transduced cells when compared to nontransduced cells, suggesting that the antibody bound phosphorylated forms of H2AX do not perturb the subsequent recruitment and accumulation of RSR and DNA repair proteins.

Using our newly developed tools, we have investigated the intranuclear kinetics of γ-H2AX accumulation and its relevance for human cell viability. It is widely accepted that γ-H2AX causes an alteration in chromatin structure that facilitates DNA repair upon genotoxic insults. Although several kinases seem to be implicated in H2AX phosphorylation, numerous studies have shown that ATM is mainly responsible for this process and this allows the precise orchestration of the recruitment of many components of the repair machinery to DSBs. Nevertheless, the accumulation of γ-H2AX over megabase domains at the DSB sites is not fully understood, but can microscopically be visualized as discrete foci [36]. This H2AX phosphorylation process is thus considered as a central player in DDR and RSR [37]. Recently, it has been shown that the foci recorded under these conditions correspond to clusters of nanofoci that were suggested to surround the DSB sites [23]. In contrast, less clear is the formation of widespread nuclear γ-H2AX patterns when the cells are continuously challenged with genotoxins and RSR inhibitors. Ewald et al. [25] proposed that the intense pan-nuclear location of γ-H2AX observed after checkpoint abrogation may signal the altered DNA structures throughout the nucleus. More recently, it was suggested that pan-nuclear formation of the γ-H2AX pattern is elicited by diffusion of active kinases from DSBs [17]. In agreement, we have found that DNA-PK, which is involved in NHEJ repair, can mediate the widespread homogenous H2AX phosphorylation. Since no extensive DNA breakage could be evidenced when this event occurs (Supplementary Figure S6), it begs the question of how can the activity of DNA-PK be triggered homogenously in the nucleus at large distances from the sites of DNA damage. Moreover, we have also observed that pan-nuclear staining of γ-H2AX is accompanied by extensive hyperphosphorylation of RPA located at the ss-DNA structures generated at the stalled forks (Figure 5) [38]. Several reports have found that RPA hyperphosphorylation is mediated by DNA-PK that, in turn, may either facilitate DNA repair [39] or promote the cellular commitment to apoptosis [40,41]. It is conceivable that H2AX in the proximity of stalled forks becomes incidentally phosphorylated together with RPA by activated DNA-PK, in addition to γ-H2AX foci located in proximity of DSBs. Pan-nuclear H2AX could thus be an indicator of global halted replication and fork collapse in the nucleus. As global fork collapse cannot be rectified, it would activate cell death. Notably, we systematically observed that, soon after treatment with replication inhibitors (and not with RSR inhibitors such as V or A), the nuclei of the treated cells were enlarged suggesting that the accumulation of halted forks could modify the chromatin structure. This was particularly visible with the nonadherent HL60R cell line (Supplementary Figure S4B). In a previous work we have observed a similar nuclear swelling and rise of pan-γ-H2AX pattern in cancer cells transduced with anti-PCNA or anti-DNA polymerase alpha inhibitory Fabs [19]. It is conceivable that the chromatin relaxation that is likely mediated by the action of numerous activated histone-modifying enzymes [42] and/or which reflects excessive DNA replication origin firing [13], would allow preferential access of DNA-PK to H2AX-containing nucleosomes. DNA-PK has been qualified as being hyperactivated under such conditions of RS [16]. Moreover, the fact that the different drugs used in this study induced similar effects in both wild type and *H2AFX^−/−^* HeLa cells suggests that pan-nuclear γ-H2AX is not required for the response to intense RS generated by global DNA replication arrest [43] and the consequent death of HeLa cells. 

It is well known that, when replication is blocked, several cellular checkpoints are activated to provide time for the cell to repair the damage. Interestingly, we found that formation of pan-nuclear γ-H2AX formation was correlated with the strength of the RS-triggering effects of the different drug combinations used. Our observations are in good agreement with previous findings showing that abrogation of Chk1 kinase sensitizes the cells treated with G and causes a significant increase in H2AX phosphorylation and toxicity [25,44,45]. The same effect was also observed when targeting both ATR and Chk1 kinases [13]. We found that the combined treatment of drugs that inhibit replication, ATR and Chk1 at the same time was the most deleterious treatment, but it remains unclear which exact mechanisms govern the decision to die upon such synergistic genotoxic insults In this context, the latest cellular events preceding cell death are generally termed replication catastrophe [8], mitotic catastrophe [46], or even mitotic disaster [47], when the nuclei appear to be fragmented under the microscope before cell death. Such aberrant mitotic cells, which mirror abrogation of the G2 DNA damage checkpoint, were also observed during our γ-H2AX analyzes (Figure 2, Figure 3 and Figure 6). The disrupted nuclei in such cases were highly γ-H2AX-positive, meaning that they contained pan-nuclear γ-H2AX before being committed to death [47]. Under such lethal RS conditions, it would be of interest to analyze how cell lines containing p53 protein (such as U2OS) would manage the balance between delayed survival and death [2,48] Overall, it is clear from this study that the stressed cells cannot recover when pan-nuclear γ-H2AX is formed and that, at this state, irreversible cell death is initiated. This is supported by the proposal that in fibroblast cell lines UV-induced pan-nuclear γ-H2AX is a preapoptotic signaling event [49]. Whether high levels of pan-nuclear γ-H2AX are generated in patients [50] or in human tumor samples [1] following administration of cytotoxic doses of chemotherapeutics remains to be established. Thus, similarly to hyperphosphorylated RPA, which is now considered as a marker of ssDNA at the stalled forks [38], we propose that pan-nuclear γ-H2AX represents a surrogate marker of RS-induced cell death.

## 4. Materials and Methods

### 4.1. Antibodies

The anti-γ-H2AX monoclonal antibodies were generated as described [19]. Briefly, BALB/c mice were immunized with the synthetic phosphorylated peptide CKATQA(p)SQEY corresponding to the C-terminus of H2AX after covalent cross-linking to ovalbumin. The hybridomas were screened by testing the culture supernatants by ELISA with both immunizing and nonphosphorylated peptides on plate and by cell staining using fixed HeLa cells treated with HU. The selected monoclonal antibodies were purified from hybridoma supernatant on Protein G Sepharose (GE Healthcare, Vélizy-Villacoublay, France) and kept at 4 °C in PBS at a concentration above 5 mg/mL. The Fab fragments of clone 3F4 were obtained by digesting pure antibody samples with papain (Sigma-Aldrich, St-Quentin Fallavier, France) as described [51], followed by size-exclusion chromatography on Superdex S200 10/300 (GE Healthcare). They were subsequently concentrated with centrifugal filtration units (Merck Millipore, Molsheim, France) to obtain Fab samples of approximately 5 mg/mL. Labeling of the Fab was performed by incubating purified Fab fragments (2.5 mg/mL) with Alexa Fluor^TM^ 488 succinimidyl ester (Invitrogen, Carlsbad, CA, USA) at a ratio 1:4 during 1 h at room temperature. Unreacted fluorophore molecules were removed using a desalting spin column (Thermo Fisher Scientific, Illkirch, France). The labeled Fab molecules in the flow-through fraction were frozen down in the presence of 10% glycerol and kept at −80 °C until use in the transduction experiments. Anti-phospho-H2AX monoclonal antibody JBW301 was obtained from Merck-Millipore. Anti-53BP1 and anti-actin rabbit polyclonal antibodies were obtained from Sigma-Aldrich. The anti-phospho-RPA (S4/S8) rabbit polyclonal antibody was obtained from Bethyl Laboratories (Montgomery, OH, USA). The phospho-Chk1 polypeptide was detected with rabbit monoclonal antibody 133D3 (Cell Signaling Technology, St-Quentin-en-Yvelines, France).

### 4.2. Cell Culture and Assays

The HeLa, U2OS, H1299, and HFF-1 cells (laboratory stocks) were maintained in Dulbecco’s Modified Eagle’s Tissue Culture Medium (DMEM; Life Technologies, Carlsbad, CA, USA) at 37 °C in a humidified 5% CO_2_ atmosphere. The HL60R [27] cells were grown in Roswell Park Memorial Institute medium (RPMI-1640; Life Technologies) under similar conditions. Both media were supplemented with L-glutamine (2 mM), gentamicin (50 μg/mL), and 10% heat-inactivated fetal calf serum. Fresh cells were thawed from frozen stocks after 10 passages. Transduction experiments with purified antibodies or Fab by electroporation were performed as previously described [21,32]. Where indicated, the cells were treated with hydroxyurea (HU; 2 mM), gemcitabine (G; 0.1 μM), VE-821 (V; 1 μM), or AZD-7762 (A; 0.1 μM), or combinations of these drugs at the same concentration. For some experiments, we added either KU-60019 (K; 10 μM) or NU-7441 (N; 5 μM) to the drug mixture. Alternatively, the cells were treated with epirubicin (0.5 μM) or camptothecin (1 μM). All drugs were purchased from Sigma-Aldrich. The number of cells remaining attached to the dish after drug treatment was determined by manual counting after dissociation with trypsin and staining with Trypan blue or by spectrometry with the PrestoBlue^TM^ cell viability kit (Life Technologies) according to manufacturer’s protocol. Before staining with 0.1% crystal violet the treated cells were fixed with 4% paraformaldehyde for 15 min. Where indicated, the harvested cells were also subjected to genomic DNA extraction. Typically, 10^5^ cells in 200 mM Tris-HCl pH 8, 5 mM EDTA, 10 mM NaCl, 1% SDS, and proteinase K (200 μg/mL, Roche Life Science, Indianapolis, IN, USA) were incubated for 5 h at 55 °C and, after addition of DNAse-free RNAseA (Sigma-Aldrich; 5 μg/mL), the samples were treated twice with phenol:chloroform:isoamyl alcohol (25:24:1). Extracted DNA was concentrated with ethanol and analyzed by agarose gel (0.8%) electrophoresis. Single cell gel electrophoresis assays were performed with the CometAssay^TM^ kit (Trevigen, Gaithersburg, MD, USA) according to manufacturer’s protocol. 

### 4.3. Generation of the H2AFX^−/−^ Cell Lines

The plasmid pCas9-2A-Puro (Addgene, Cambridge, MA, USA) was digested with BpiI restriction enzyme to insert the annealed oligonucleotides 5′- CACCGCGGGCCCTCTTAGTACTCC-3′ and 5′-AAACGGAGTACTAAGAGGGCCCGC-3′. The resulting construct was transfected in HeLa cells using jetPRIME (Polyplus Transfection, Illkirch, France). The transfected cells submitted to a puromycin (0.2 µg/mL) selection for 3 days [52] and further allowed to grow for 2 weeks in the absence of the antibiotic. Single cell clones were isolated and cells of several clones were analyzed by Western blotting using the monoclonal antibodies described in this study to confirm the absence of H2AX expression. The detailed analysis of these cell lines will be published elsewhere. 

### 4.4. Western Blot and ELISA

For the analysis of the U2OS proteins, soluble extracts (60 μg/lane) in RIPA buffer were used. γ-H2AX and actin were revealed with monoclonal antibody 3F4 (0.2 µg/mL) and rabbit polyclonal serum A2066 (Sigma-Aldrich), respectively. Bound secondary HRP-labeled antibodies were revealed with ECL reagent (GE Healthcare) and analyzed with the Image QuantLAS 4000 imager (GE Healthcare). For the ELISA assays, microtiter wells (Thermo Fisher Scientific) were coated with 2 μg/mL of phosphorylated or nonphosphorylated peptide CKATQASQEY in PBS overnight at 4 °C. The tested monoclonal antibodies or Fab fragments were diluted in PBS containing 0.1% NP40 and following incubation at RT for 1 h they were revealed with sheep HRP conjugated anti-mouse IgG (GE Healthcare). After several washes with PBS containing 0.1% NP40 and addition of 3′,3′,5′,5′-tetramethylbenzidine (Sigma-Aldrich), the optical density was measured at 450 nm in an ELISA reader. 

### 4.5. Immunofluorescence Microscopy

For the analysis by classical immunofluorescence microscopy, the cells were fixed with 4% paraformaldehyde for 15 min and, after permeabilization with 0.2% Triton X 100 for 5 min, they were incubated with different antibodies diluted in PBS containing 10% fetal calf serum. The primary antibodies were detected with Alexa Fluor 488 or 568 labeled-anti-mouse or anti-rabbit immunoglobulins (Life Technologies). After incubation, the coverslips were mounted with 4′,6′-diamino-2phenyl-indole (DAPI) Fluoromount-G (Southern Biotech, Birmingham, AL, USA) and imaged with a Leica DM5500 microscope (Leica, Wetzlar, Germany) equipped with 63× and 100× objectives. The signal was recorded on an EM-CCD camera (Hamamatsu, Massy, France). Live-cell time-lapse experiments were performed with a Leica DMIRE 2 microscope equipped with a control system of 37 °C, 5% CO_2_ (Life Imaging Services, Basel, Switzerland), a 40× HXC PLAPO PH3 (1.25 NA) objective. Images were acquired with a Photometrics Prime sCMOS camera piloted by the Metamorph software allowing phase-contrast imaging. Fluorescence images were recorded by using a Xenon-lamp and Leica L5 filter cube. Confocal microscopy and three-dimensional structured illumination microscopy (3D SIM) were performed as previously described [32]. All microscopy images were processed using the Fiji/Image J software [53]. For the calculation of the proportion of pan-nuclear cells, the images of ten microscopy fields acquired with the 40× objective were used. For the measurement of the nuclear fluorescence intensity, the nuclei were set with the DAPI channel acquisition as regions of interest (ROI) and the mean fluorescence intensity in each ROI was measured using the Fiji built-in tool. The mean values and the Pearson’s standard deviations were calculated with the Excel software (Microsoft, Issy-les-Moulineaux, France).

## 5. Conclusions

This study shows that H2AX is increasingly phosphorylated upon sustained treatment with combined drugs that provoke RS and that the levels of γ-H2AX culminate and become easily visible in the whole nucleus before cell death. Moreover, our results show in HeLa cells that H2AX is not required for RS-induced cell death, but can be considered as a consequence of the strength of the RS-induced cell death. In agreement with Parsels et al. [15], our study demonstrates that using a highly specific anti-γ-H2AX mAb allows the detection of the widespread, pan-nuclear γ-H2AX pattern that is proportional to RS-induced cell death. As the pan-nuclear γ-H2AX pattern is easily distinguishable from the focal γ-H2AX pattern, the pan-nuclear γ-H2AX pattern will be extremely useful to determine the efficacy of chemotherapeutic drug combinations to kill tumor cells in translational studies.

## Supplementary Materials

The following are available online at https://www.mdpi.com/2072-6694/11/3/355/s1, Figure S1: Binding properties of the anti-γ-H2AX monoclonal antibodies used in this study, Figure S2: Detection of y-H2AX upon prolonged incubation of HeLa cells with HU, Figure S3: Dynamics of γ-H2AX levels in U2OS and H1299 cells, Figure S4: Dynamics of γ-H2AX formation in HeLa and HL60R cells, Figure S5: Detection of γ-H2AX after treatment with epirubicin and campthotecin, Figure S6: Detection of γ-H2AX following cotreatment with the DNA-PK inhibitor and analysis of the genomic DNA integrity, Figure S7: Cell death induction upon irreversible formation of widespread nuclear γ-H2AX, Figure S8: Importance of pan-nuclear γ-H2AX formation for cell death, Figure S9: Binding specificity of the 1H2 and 3F4 Fabs as probed by ELISA, Video S1: Time-lapse monitoring of transduced U2OS cells. 24 h post-transduction with Alexa Fluor 488-labeled 3F4 Fabs, the cells were subjected to phase-contrast microscopy and images were taken every 30 min for 8 h. Magnification: 400×, Video S2: Time-lapse monitoring of transduced U2OS cells after treatment with G+V. The cells were processed as indicated in the legend of Video S1, except that G+V drugs were added in the medium at 12 h post-transduction. Time-lapse analysis was started at 46 h post-transduction and carried out as indicated in the legend of Video S1, Magnification: 400×.
