# Supplementary material for: Uniform Widespread Nuclear Phosphorylation of Histone H2AX Is an Indicator of Lethal DNA Replication Stress
Source: Cancers (Basel). 2019 Mar 13;11(3):355. doi: 10.3390/cancers11030355 (PMC6468890; doi:10.3390/cancers11030355)
Supplement: Supplementary file 1 [file cancers-11-00355-s001.zip › cancers-433513-supplement-final-.docx]

Supplementary Materials

Uniform Widespread Nuclear Phosphorylation of Histone H2AX Is an Indicator of Lethal DNA Replication Stress

Eric Moeglin, Dominique Desplancq, Sascha Conic, Mustapha Oulad-Abdelghani, Audrey Stoessel, Manuela Chiper, Marc Vigneron, Pascal Didier, Laszlo Tora and Etienne Weiss


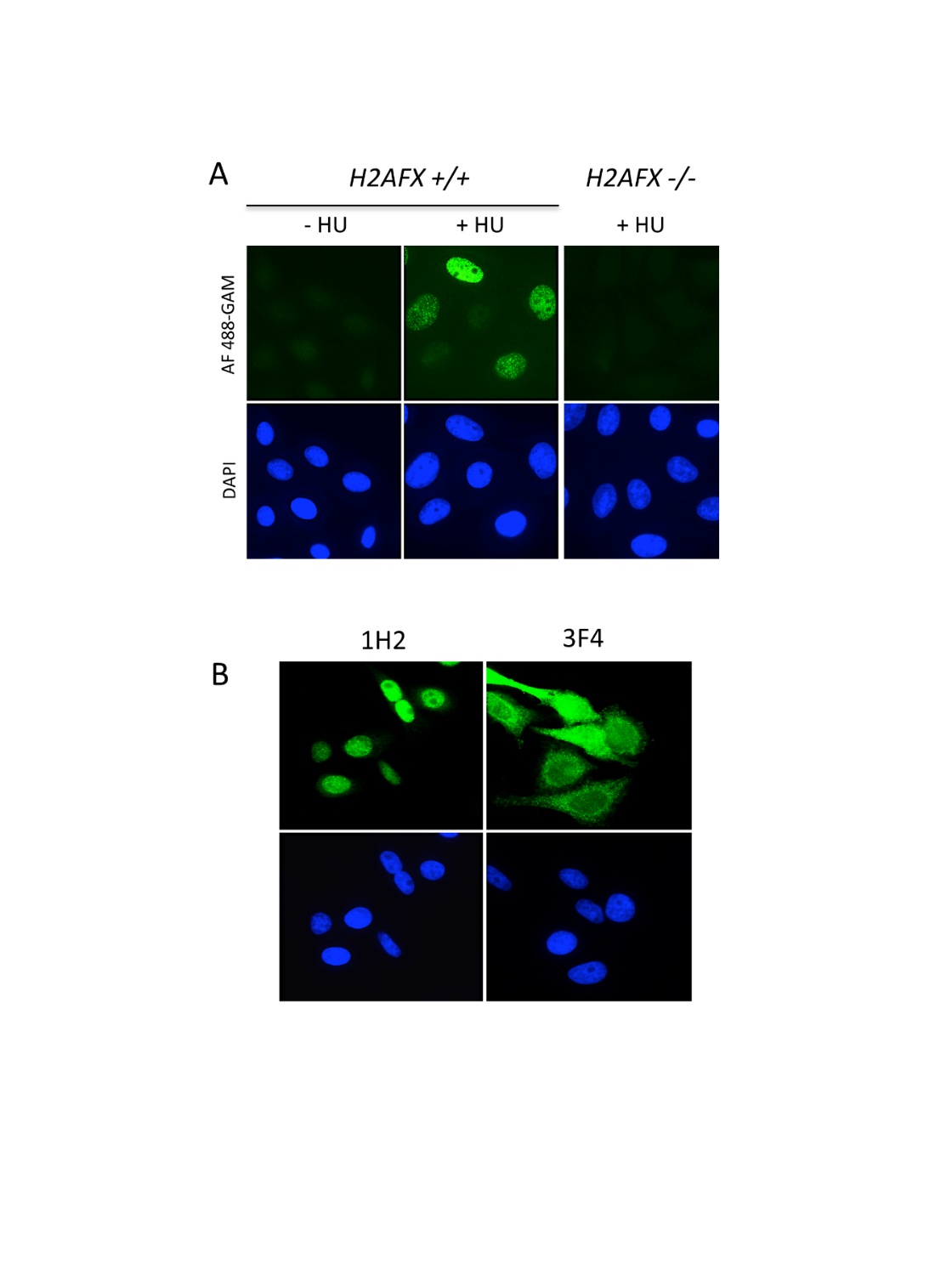


**Figure S1.** Binding properties of the anti-γ-H2AX monoclonal antibodies used in this study. (**A**) Immunofluorescence detection of γ-H2AX with the commercially available JBW301 antibody in HU-treated wild-type or H2AFX^−/−^ HeLa cells. (**B**) Behavior of mAbs 1H2 and 3F4 in HeLa cells after intracellular delivery by electroporation. The delivered antibodies were revealed with Alexa Fluor 488-labelled anti-mouse globulins 24 h post-transduction. Magnification: 630×.


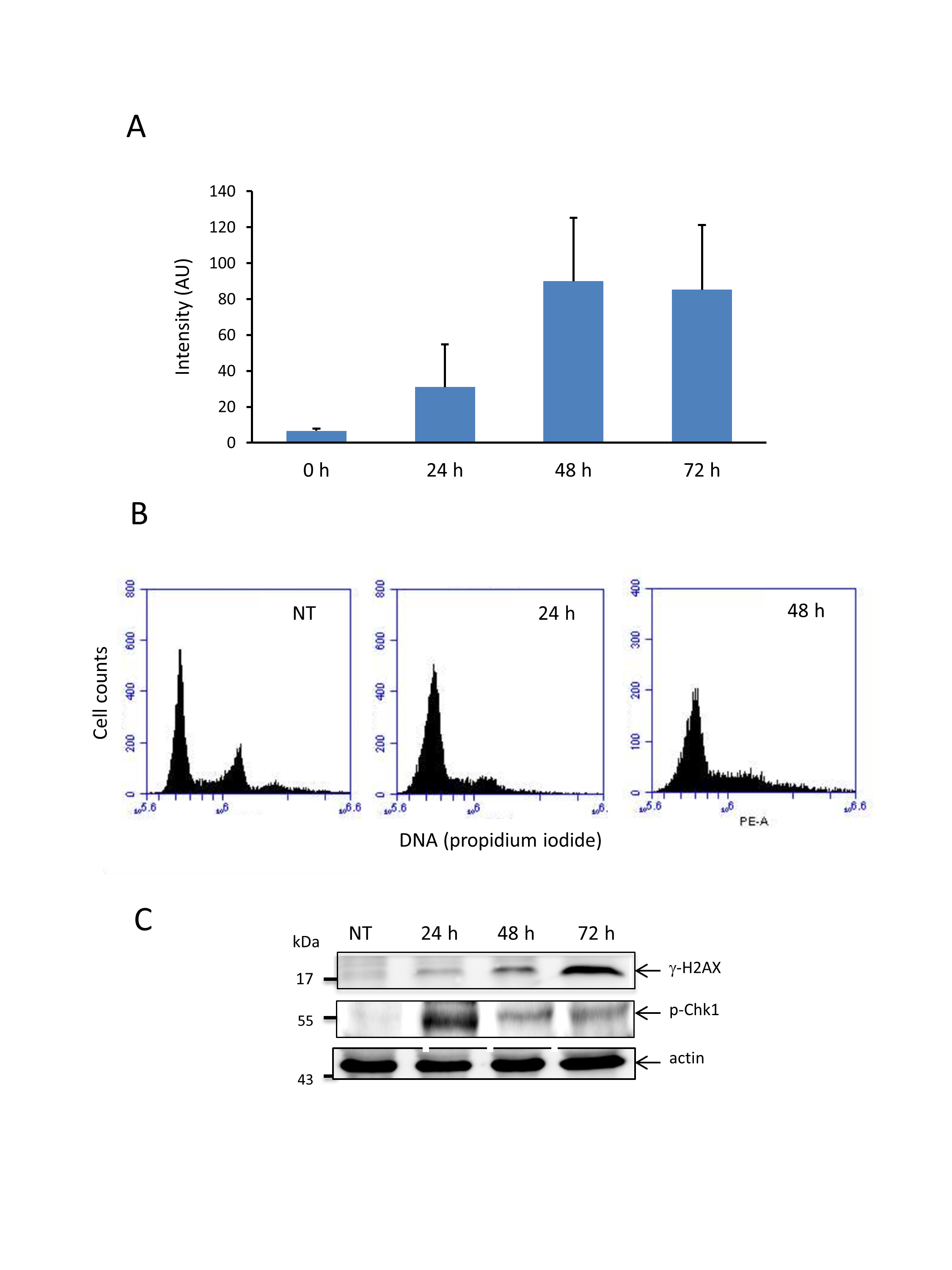


**Figure S2.** Detection of y-H2AX upon prolonged incubation of HeLa cells with HU. (**A**) The histogram corresponds to the average nuclear fluorescence intensity of a minimum of 100 nuclei per time point as shown in Figure 1E. (**B**) HU-treated HeLa cells as in A were harvested at the indicated time points and subjected to FACS analysis after propidium iodide staining. Approximately 2 × 10^4^ cells were counted in each case. The panels correspond to the measurement of the DNA content (cycling state). (**C**) Binding of mAb 3F4 as assayed by Western blotting. HeLa cells (5 × 10^4^) were incubated with HU and crude extracts (30 μg) prepared at the indicated time points were subjected to acrylamide gel electrophoresis and blotting. The indicated polypeptides were probed with relevant antibodies. Bound 3F4 antibody and anti-pChk-1 antibodies were revealed with HRP-labelled anti-mouse or anti-rabitt globulins, respectively. Actin was used as a loading control. The migration of molecular weight markers is indicated.


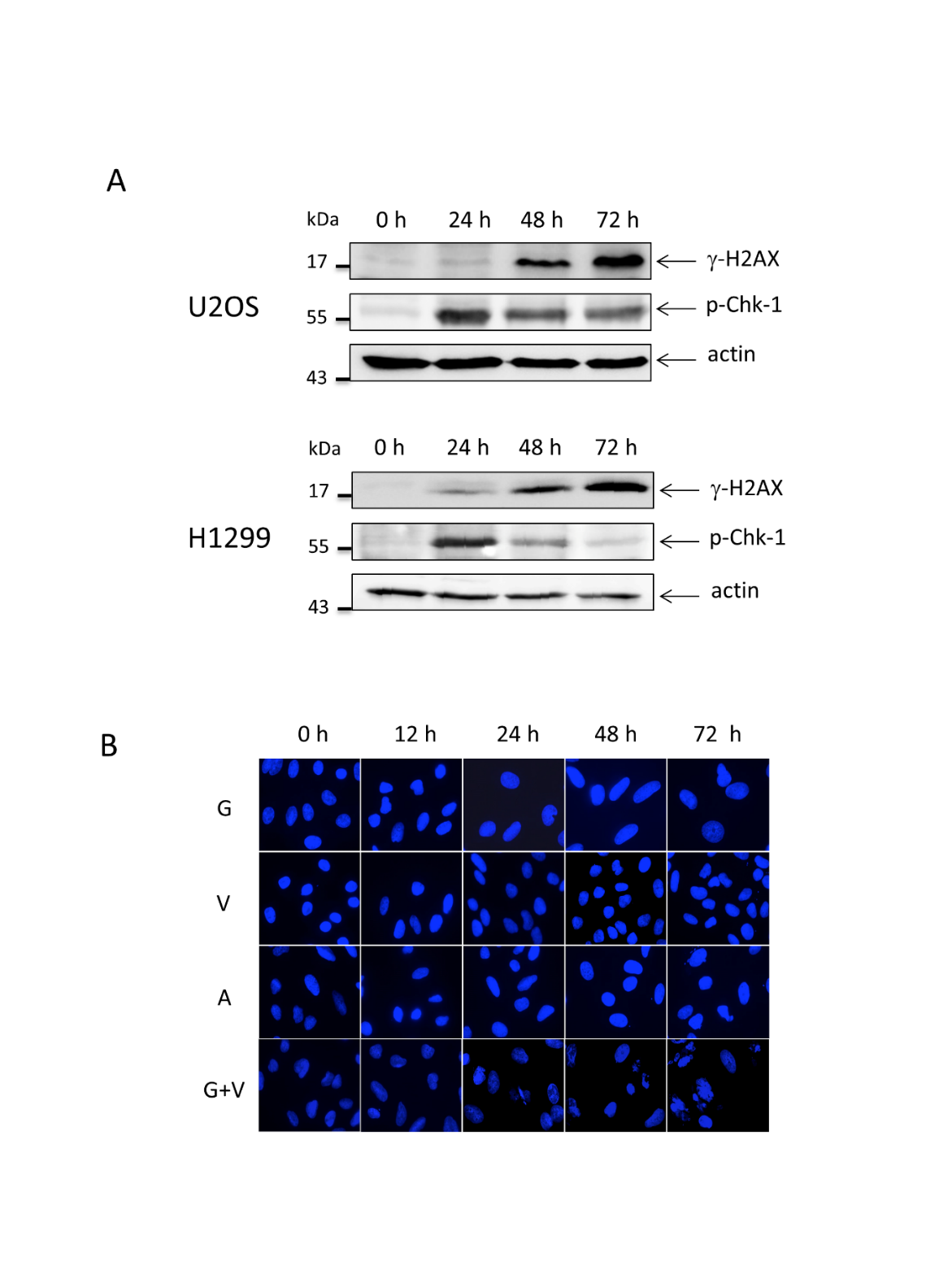


**Figure S3.** Dynamics of γ-H2AX levels in U2OS and H1299 cells. (**A**) Analysis of the accumulation of γ-H2AX and phospho-Chk-1 polypeptides upon HU treatment. Crude cell extracts (35 μg) were prepared at the indicated time points and subjected to Western blotting as described in the legend of Supplementary Figure S2. (**B**) The pictures correspond to the cells represented in Figure 4A after counterstaining with DAPI. Magnification: 630×.


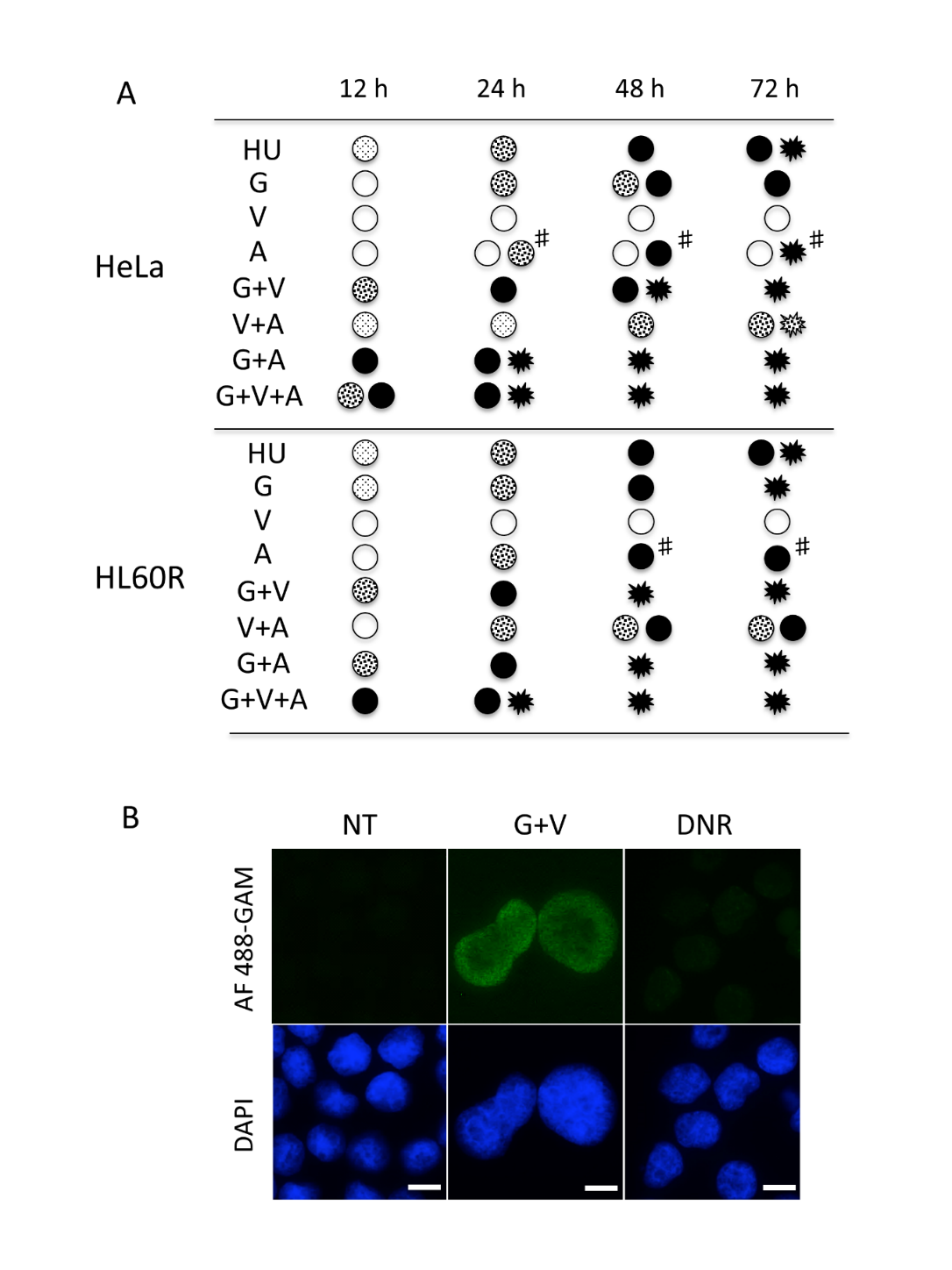


**Figure S4.** Dynamics of γ-H2AX formation in HeLa and HL60R cells. (**A**) The γ-H2AX levels measured by immunofluorescence after treatment with the indicated drug combinations are represented as symbols, as described in Figure 3. (**B**) Analysis by immunofluorescence of γ-H2AX in HL60R cells after treatment with G+V for 24 h. The staining of γ-H2AX was performed as indicated in the legend of Figure 2. Scale bar: 10 μm. DNR, daunorubicin.


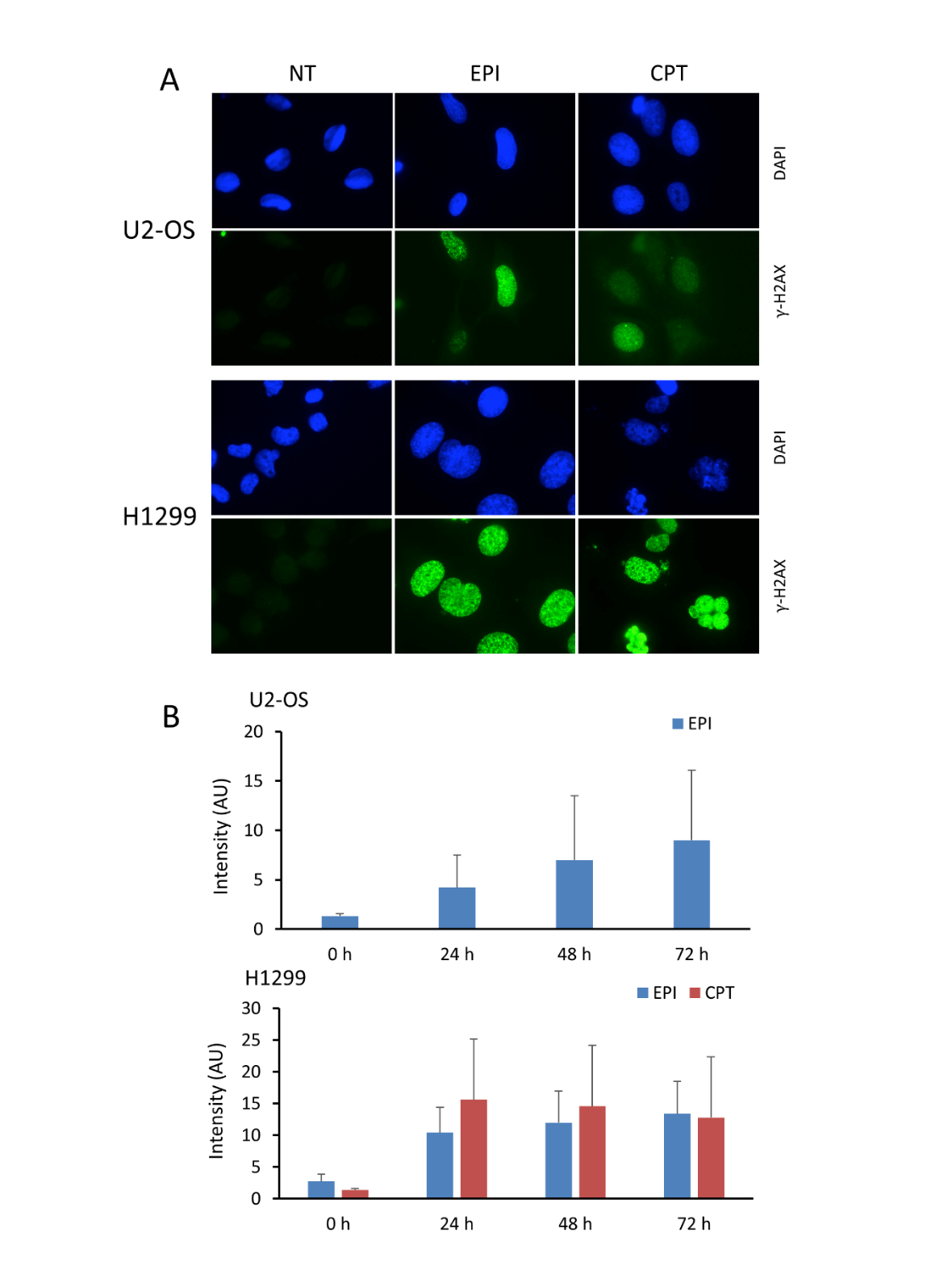


**Figure S5.** Detection of γ-H2AX after treatment with epirubicin and campthotecin. U2OS and H1299 cells were treated with epirubicin (EPI) or campthotecin (CPT) (Materials and Methods) and the formation of widespread nuclear γ-H2AX was monitored by immunofluorescence with mAb 3F4 24 h, 48 h and 72 h post-treatment. (**A**) The micrographs show typical fields of cells observed under the microscope 72 h post-incubation after counterstaining with DAPI. Magnification: 630×. (**B**) The fluorescence intensity of the nuclei of at least 150 cells from two independent experiments after treatment with either EPI or CPT (H1299) or EPI (U2OS) was calculated. The mean values obtained at the indicated time points are represented.


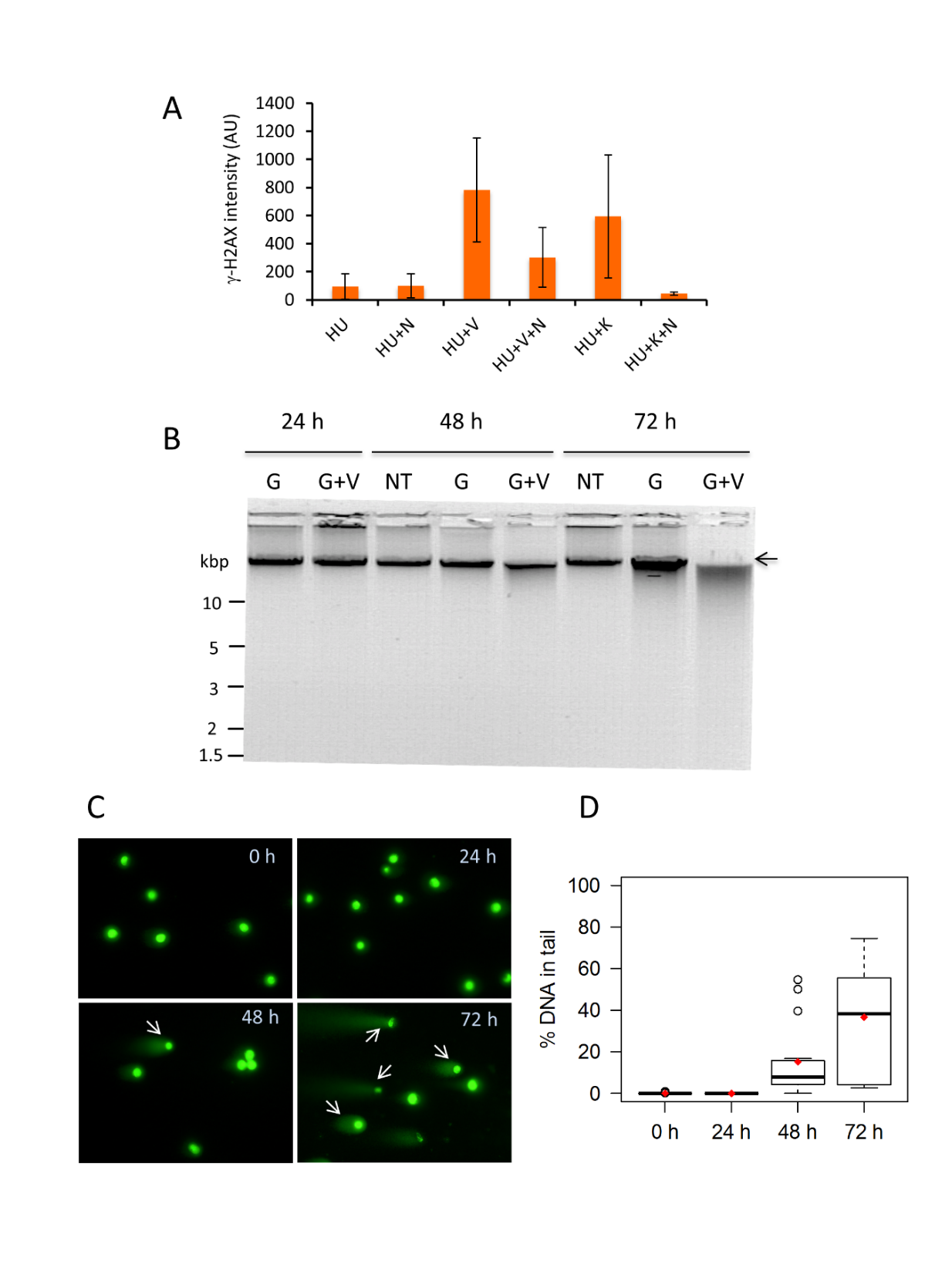


**Figure S6.** Detection of γ-H2AX following co-treatment with the DNA-PK inhibitor and analysis of the genomic DNA integrity. The graph shown in (**A**) represents the mean values of the nuclear fluorescence intensity of 125 U2OS cells treated as indicated. (**B**) U2OS cells were treated as indicated and their genomic was extracted at 24 h, 48 h and 72 h post-treatment. Samples of 5 × 10^4^ cells containing a similar amount of DNA were loaded on agarose gel. The DNA bands were visualized by UV-illumination following staining with ethidium bromide. DNA markers were migrated in parallel. NT, non-treated. (**C**) U2OS cells treated with G+V for 24 h, 48 h or 72 h were assayed by single cell gel electrophoresis (Comet assay; Materials and Methods) and subsequently analyzed by fluorescence microscopy after staining with SYBR Gold. The micrographs correspond to typical fields observed with a 20× objective. The comet tails are indicated (arrows). (**D**) Quantitative analysis of DNA present in the comet tail of the U2OS cells treated as in C. Up to 50 cells/time point were used for calculating the ratios (expressed in %) between the signal of DNA present in the comet tail and the signal of DNA of the whole cell.


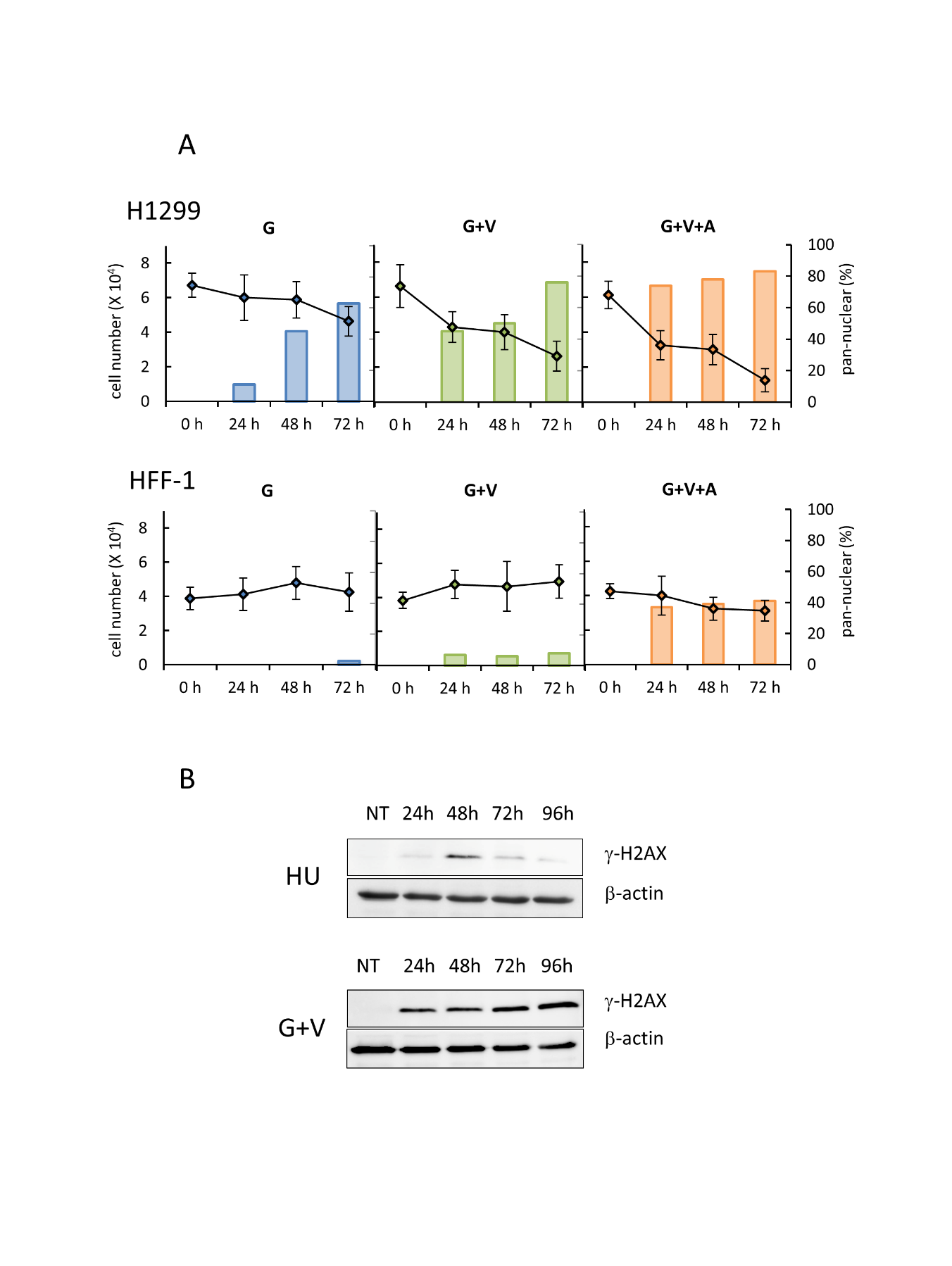


**Figure S7.** Cell death induction upon irreversible formation of widespread nuclear γ-H2AX. (**A**) Time course analysis of the percentage of pan-nuclear γ-H2AX stained H1299 or HFF-1 cells after treatment with G, G+V or G+V+A. The curves correspond to the number of cells that remained attached to the plastic in experiments done in parallel. A minimum of 150 cells were analysed in each condition. (**B**) Variation of γ-H2AX levels in U2OS cells treated for 24 h (pulse treatment) with the indicated drugs as probed by Western blotting with mAb 3F4. Following treatment, the U2OS cells (5 × 10^4^) were incubated in fresh medium and extracts (30 μg) were prepared at the indicated time points, as indicated in the legend of Figure 5B. Actin was used as a loading control.


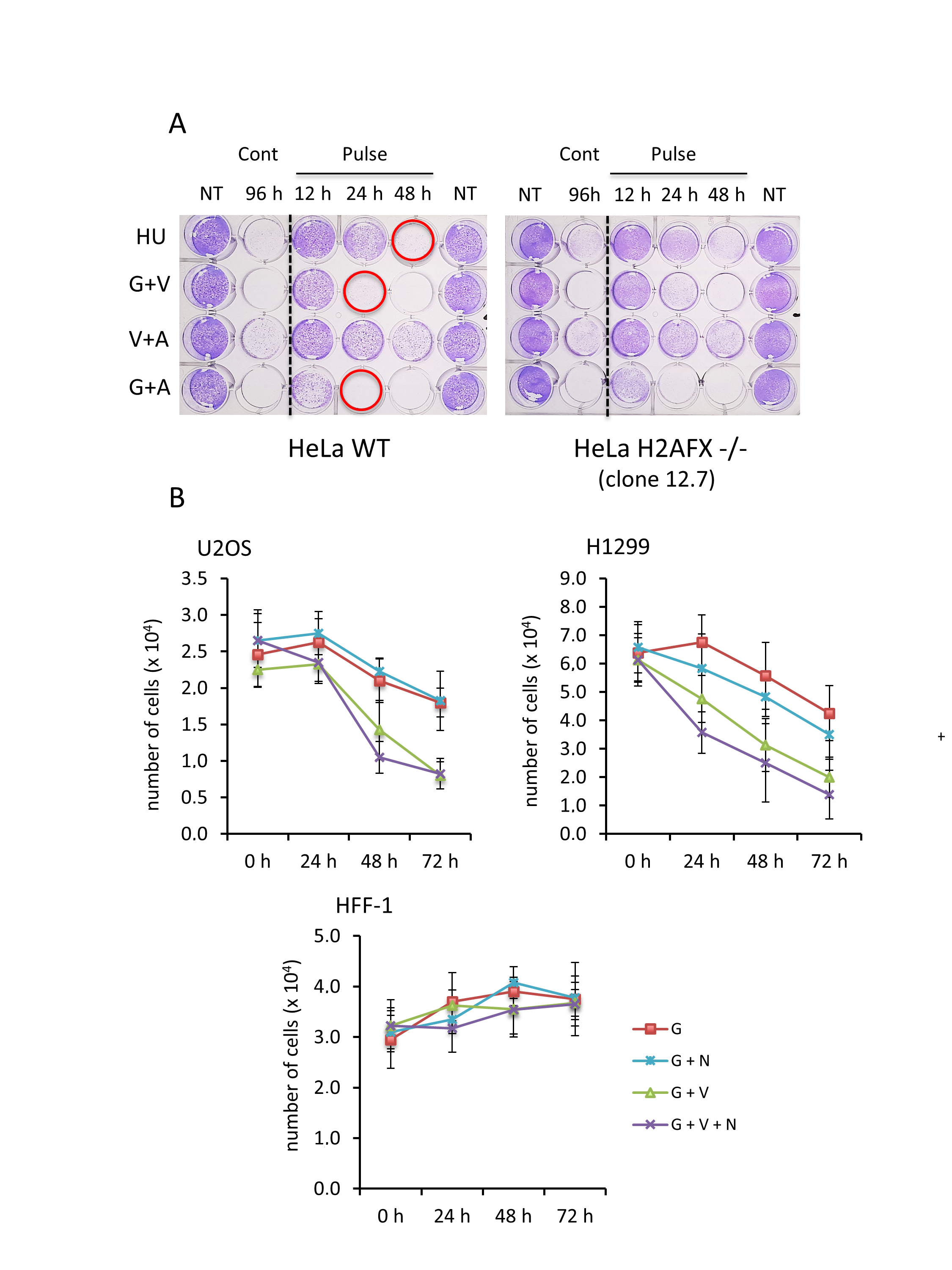


**Figure S8.** Importance of pan-nuclear γ-H2AX formation for cell death. (**A**) The represented viability assays were performed with either wild-type (WT) or *H2AFX*^−/−^ HeLa cells as described in the legend of Figure 6D. (**B**) U2OS, H1299 and HFF-1 cells were treated with G (red), G+N (blue), G+V (green) and G+V+N (purple) and their survival rate was analysed over a total period of 72 h by counting the cells remaining bound to the plastic. The graphs correspond to the number of viable cells counted at 0 h, 24 h, 48 h and 72 h post-treatment.


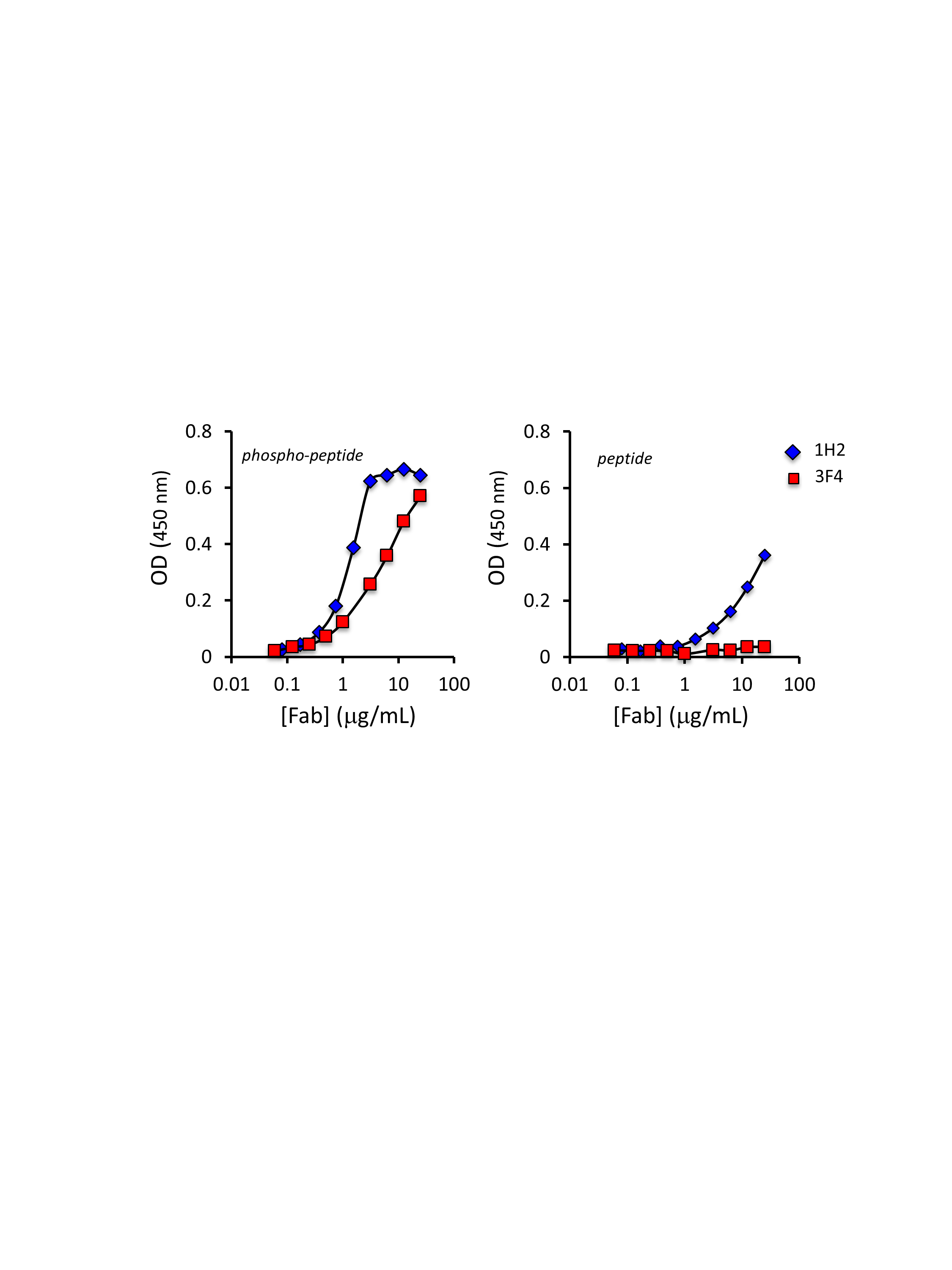


**Figure S9.** Binding specificity of the 1H2 and 3F4 Fabs as probed by ELISA. Varying amounts of purified Fabs were allowed to react with either the phosphorylated (*phospho-peptide*) or the non-phosphorylated (*peptide*) peptides corresponding to the C-terminus of H2AX coated on plate. Bound material was revealed with HRP-labelled secondary anti-mouse globulins. The color code is indicated.

| 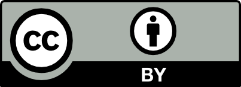 | © 2019 by the authors. Licensee MDPI, Basel, Switzerland. This article is an open access article distributed under the terms and conditions of the Creative Commons Attribution (CC BY) license (http://creativecommons.org/licenses/by/4.0/). |
| --- | --- |
